# Construction of a novel model based on PVT1-MYC duet-related genes for predicting survival and characterization of the tumor microenvironment in pancreatic cancer

**DOI:** 10.3389/fimmu.2024.1435593

**Published:** 2024-09-23

**Authors:** Bo Ren, Jie Ren, Minzhi Gu, Xiaohong Liu, Lei You, Yupei Zhao

**Affiliations:** ^1^ Department of General Surgery, Peking Union Medical College Hospital, Peking Union Medical College, Chinese Academy of Medical Sciences, Beijing, China; ^2^ Key Laboratory of Research in Pancreatic Tumor, Chinese Academy of Medical Sciences, Beijing, China; ^3^ National Science and Technology Key Infrastructure on Translational Medicine in Peking Union Medical College Hospital, Beijing, China

**Keywords:** pancreatic cancer, PVT1-MYC duet, prognosis, tumor microenvironment, CDC6

## Abstract

Pancreatic cancer is an extremely malignant tumor. PVT1 and MYC signaling has been considered as a therapeutic target recently. Nonetheless, the prognostic values and critical regulatory networks of PVT1-MYC duet in pancreatic cancer remain unclear. Firstly, we identified PVT1-MYC duet-related genes using public databases. Then we analyzed our Hi-C and ChIP-seq data to confirm PVT1-MYC duet. We performed LASSO regression and multivariate Cox regression analysis to build a prognostic model whose effectiveness and robustness were validated by Cox regression, ROC analysis, calibration curve, and nomogram. Besides, we conducted functional enrichment analyses, mutation profiles analyses and the immune features analyses to compare low- and high-risk group. Functional enrichment analyses revealed that several terms associated with cancer progression were enriched in the high-risk group. Mutation profile analysis showed that high-risk group had higher tumor mutation burden, and immune analysis demonstrated high-risk group had more immunosuppressive tumor microenvironment. Finally, we detected PVT1 expression in pancreatic cancer and paracancer tissues from the PUMCH cohort, which showed that PVT1 was significantly upregulated in pancreatic cancer and associated with invasion, metastasis, and poor prognosis. We further performed transwell and proliferation assays and found that PVT1, CDC6, and COL17A1 could promote migration or proliferation of PDAC cells. This study constructed a prognostic model based on three PVT1-MYC duet-related genes, which had a significant potential in predicting the prognosis and tumor microenvironment of pancreatic cancer. These results suggested that targeting PVT1-MYC duet or its regulatory processes could be a therapeutic option with great interests.

## Introduction

Pancreatic cancer is the fourth most common cause of cancer death in the United States. It is a major cause of cancer-associated mortality, with a dismal overall prognosis that has remained virtually unchanged for many decades. Pancreatic ductal adenocarcinoma (PDAC) is the most common type of pancreatic cancer featured with high intra-tumoral heterogeneity and poor prognosis. Despite tremendous efforts, PDAC is still related to a short survival with about 9% five-year survival rate now. Although surgery remains almost the only option for patients with PDAC to obtain long term survival, 5-year survival rate of patients who undergo surgical resection is only 30% (1). Serum carbohydrate antigen 199 (CA199) (2) and TNM staging (3) are used to predict prognosis of PDAC. To evaluate the prognosis more accurately, the molecular markers are considered as the new research direction. Therefore, it is necessary to find new prognostic markers of PDAC, which may facilitate a breakthrough in its precision medicine to guide PDAC treatment in the future.

Plasmacytoma variant translocation 1 (PVT1) is a lncRNA encoded by Pvt1 oncogene locating at chromosome 8q24.21, which was first identified in human cancer translocations as a recurrent breakpoint in Burkitt’s lymphoma (4, 5). It has been demonstrated that PVT1 is important for multiple types of cancer progression (6–8). For instance, PVT1 could interact with EZH2 to guide PRC2 to suppress expression of genes associated with pro-apoptotic and tumor suppressor, to promote multiple myeloma progression (9). Meanwhile, PVT1 could bind with TAZ protein to prevent its phosphorylation, which promotes stemness of renal carcinoma (10). Furthermore, inhibition of PVT1 could promote CD8^+^ T cells infiltration and metastasis of head and neck squamous cell carcinoma (11). Besides, our previous work demonstrated that PVT1 is associated with PDAC chemoresistance. Gemcitabine can trigger lncRNA PVT1 to its encoded miRNAs, such as miR-1207 pair that enhanced PDAC chemosensitivity by inhibiting SRC proto-oncogene and Ras homolog family member A in PDAC cells (12). Similarly, the oncogene MYC is also upregulated and activated in PDAC, inducing vital cellular processes to promote progression of PDAC. Ravikanth et al. found that MYC levels, including gene amplification and transcriptional upregulation, were positively associated with metastatic burden of PDAC, due to recruitment of tumor-associated macrophages (13). Our previous work also demonstrated that guanidinoacetic acid anabolism could upregulate MYC via active histone modifications (14), and MYC could upregulate HMGA2 to promote PDAC metastasis (15). These studies emphasized the important roles of PVT1 and MYC in development of PDAC.

Recently, a concept called “PVT1-MYC duet” has been raised (16). On the one hand, PVT1 and MYC locate at chromosome 8q24, which leads to co-amplification of them in various types of cancer (16). On the other hand, PVT1 could attenuates MYC phosphorylation, which stabilizes MYC protein. Thus, evaluating the status of PVT1-MYC duet is a powerful method for prognosis estimation of pancreatic cancer. However, few studies focused on the prognostic value of PVT1-MYC duet related genes and the potential targets of the duet in pancreatic cancer.

In the present study, we first use correlation analysis to identify the PVT1-MYC duet-related genes. Then we systematically analyzed the expression profiles and prognostic values of PVT1-MYC duet-related genes using public datasets to construct PVT1-MYC duet-related signature and compare functional enrichment, somatic mutation profiles, and immune features between the low- and high-risk subgroups to explore the potential regulatory mechanisms. We finally detected PVT1 expression in pancreatic cancer tissues and tumor-adjacent normal tissues by ISH method and analyzed the correlation between PVT1 expression and clinicopathological parameters, and confirmed the function of PVT1 and signature genes in the progression of PDAC. The results of this study may help to improve the current plight of the mechanism of PVT1-MYC duet in pancreatic cancer and the therapeutic strategies targeting PVT1-MYC duet-related processes.

## Materials and methods

### Datasets and processing

The RNA-seq data and corresponding clinicopathological features of pancreatic cancer and normal pancreatic tissue from the Genotype-Tissue Expression (GTEx) and The Cancer Genome Atlas (TCGA) were downloaded from the Xena (https://xenaborwser.net/datapages), including 167 normal pancreatic samples and 178 pancreatic cancer samples. The expression data was normalized by TPM method (transcripts per million) and transformed to log_2_(TPM+1) for differentially expressed genes analysis. The RNA-seq and clinicopathological data of pancreatic cancer from the Internal Cancer Genome Consortium (ICGC) were also obtained from Xena, including 96 pancreatic cancer samples. The counts data was normalized to CPM (counts per million) and transformed to log_2_(CPM) for downstream analysis. The microarray and clinical data of GSE62452 and GSE78229 datasets, including 66 and 49 pancreatic cancer samples, perspectively, were obtained from the Gene Expression Omnibus (GEO) database (http://www.ncbi.nlm.nih.gov/geo/) (**Supplementary Table 1**). To evaluate the PVT1 expression in PDAC as compared to normal tissues. We analyzed the previously published and publicly available microarray data from Oncomine database (www.oncomine.org). PVT1 expression levels are reported as Log2 median-centered intensity in the Oncomine database. The comparison of PVT1 expression between PDAC and normal tissue was conducted by the Student’s t-test to generate a P value.

Hi-C and ChIP-seq data used in this study were from our previous work (GSE149103) (17). For data processing, Hi-C data of PANC-1 and Capan-1 were processed by HiC-Pro (18) to make normalized 5-kb resolution matrices. Loops were identified by HiCCUPS module of Juicer software. Hi-C and ChIP-seq data were visualized by Juicebox (19).

### Identification of differentially expressed genes (DEGs)

We first performed spearman correlation test of RNA-seq data to find PVT1- and MYC-related genes in TCGA cohort (due to focusing on tumor-promoting genes, the cutoff value is r value > 0.3 (20, 21) and p value < 0.05), and the PVT1-MYC duet-related genes were considered as the intersection of PVT1- and MYC-related genes. Then we used the “limma” R package to identify DEGs between pancreatic cancer (TCGA) and normal pancreatic samples (GTEx). The adjusted p value < 0.05 and |log_2_(fold change)| ≥ 1 were considered as the cutoff value for identifying PVT1-MYC duet-related DEGs. The DEGs between high- and low-risk groups were also identified according to the same criteria. Visualization of DEGs was performed by volcano plots and heatmaps.

### Establishment and verification of the PVT1-MYC duet-related prognostic model

The TCGA cohort was split to the training cohort and the validation cohort. The PVT1-MYC DEGs between pancreatic cancer and normal pancreatic samples were performed least absolute shrinkage and selection operator (LASSO) regression analysis in the training cohort to further screen out the optimal gene combination (using “glmnet” R package). Then, the DEGs selected by LASSO regression were further screened to construct the best regression model via univariate and multivariate Cox regression analysis. Finally, the risk score of each sample was calculated by the multivariate Cox regression coefficient of each gene in the prognostic model with the following formula: Risk score = (Expr_gene1_ × Coef_gene1_) + (Expr_gene2_ × Coef_gene2_) + … + (Expr_genen_ × Coef_genen_). For the validation cohort and external cohorts, the risk score of each sample was calculated by the above formula. Patients in these cohorts were stratified into the high- and low-risk groups according to the median value of risk scores. Visualization was performed by the principal component analysis (PCA). The Kaplan-Meier survival analysis was used to compare the overall survival (OS) between high- and low-risk groups. The univariate and multivariate Cox regression were performed to identify the independent prognostic factors associated with OS. The nomogram, based on the result of univariate and multivariate Cox regression, was established to predict and visualize the 1-, 2-, 3-year survival probability based on the risk score and other clinicopathological features. The C-index, calibration curve and time-dependent ROC curve of 1-, 2-, 3-year were used to evaluate the predictive effectiveness of the nomogram.

### Functional enrichment analysis of DEGs between high- and low-risk groups

The ALL ontology of the DEGs between high- and low-risk groups was analyzed by Gene Ontology (GO), while the pathway enrichment was analyzed by the Kyoto Encyclopedia of Genes and Genomes (KEGG) (22). Furthermore, we performed gene sets enrichment analysis (GSEA) to find hallmarks enriched in the DEGs based on the “hallmarks” gene sets from MSigDB database (https://www.gsea-msigdb.org/gsea/msigdb). These enrichment analyses were conducted by the “clusterProfiler” R package (23).

### Somatic mutation and immune feature analysis

The landscape of somatic mutations of high or low risk samples was analyzed and visualized by the “maftools” R package. Tumor mutation burdens (TMB) were calculated by this R package and compared between the low- and high-risk groups. The estimate score, stromal score, immune score, and tumor purity were calculated by the ESTIMATE algorithm. The CIBERSORT algorithm was used to quantify the infiltration of 22 immune cells in tumor microenvironment (TME). The immune subtypes of individuals were classified by using the “ImmuneSubtypesClassifier” R package. The immunotherapy responses of individuals were analyzed by TIDE website (http://tide.dfci.harvard.edu/login/).

### Clinical specimens, tissue microarrays, and *in situ* hybridization (ISH) assay for detection of PVT1 expression

344 PDAC tissues were collected from patients who undergone radical resection of PDAC, and 298 of them had paired adjacent non-tumor tissues. All patients did not receive radiotherapy, chemotherapy, or other therapy before surgery. Both cancer and paracancer tissues were confirmed by two experienced pathologists. TMAs were constructed as described previously (24). Analyses of 642 tissues of formalin-fixed paraffin-embedded (FFPE), including PDAC tissues (n=344) and paracancer tissues (n=298), were conducted with a manual tissue arrayer (Beecher Instruments, Sun Prairie, WI). The representative cancer and paracancer tissues of each PDAC patient were punched out on two cores (diameter=1.5mm) after careful selection. PVT1 expression was detected by ISH. The ISH probe (Source: QIAGEN, Identifier: 339500LCD0164430-BKG), ISH kit (miRCURY LNA miRNA ISH Buffer set (FFPE), Source: QIAGEN, Identifier: 339450), and the anti-digoxin antibody (anti-Digoxigenin-POD, Source: Roche, Identifier: 11207733910) are used for ISH. The sequence of the ISH probe used for staining was 5’-AGCTGCAAGGTCAGTAGTGAT-3’. The 3’ and 5’ ends of the probe were labeled with digoxin to increase the signal strength. For ISH, 4 μm thick FFPE tissue sections were mounted on TMA, dewaxed in xylene, rehydrated, and then performed ISH at 50°C for 1h. After that, endogenous peroxidase was blocked by 3% hydrogen peroxidase. Subsequently, the anti-digoxin antibody was added and incubated in a water bath at room temperature for 1 hour. Sections were washed using 0.5 M phosphate-buffered saline tween and revealed with diaminobenzidine. The color-rendering results were observed under the microscope. The positive control of the probe is β-actin and the negative control is scramble. PVT1 expression was evaluated by the H-score (25), which is determined by the staining intensity and positive cell proportion. The cutoff value of PVT1 expression is the median of H-score.

### Cell culture and transfection

Cell lines BxPC-3 and T3M4 were purchased from the American Type Cultcure Collection (ATCC) and cultured with recommended medium. All cell lines were tested for Mycoplasma and identified by Short Tandem Repeat. All medium was added with 10% fetal bovine serum and 1% Penicilin-Streptomycin (Life Technologies, #15,140-122). All cell lines were cultured with 37°C and 5% CO_2_. Short interference RNAs (siRNAs) used in this study were designed and chemically synthesized by RiboBio (RiboBio, Guangzhou, China). Sequences of siRNAs were listed in **Supplementary Table 7**. For cell transfection, 5.0×10^5^ cells were transfected with 50 nM siRNA using Lipofectamine 3000 (Invitrogen, Carlsbad, CA, USA) according to the manufacturer’s instruction. At 24h or 48h post-transfection, PC cells were harvested for functional experiments.

### Cell proliferation assay

3000 cells of BxPC-3 or T3M4 were plated in the 96-wells plates containing appropriate medium with 10% FBS. The Sulforhodamine B (SRB) assay was used to evaluate cell proliferation. After fixation by 10% trichloroacetic acid and staining with 4% SRB solution, absorbance was measured at OD564 using 10nM tris-base. Six replicate wells were analyzed per group.

### Transwell assay

Transwell assays were performed as described previously (17). In brief, 5×10^4^ cells in FBS-free medium were placed into the upper chamber coated with FBS-free medium for migration. After 24h, the migrated cells were fixed and strained by the 0.1% crystal violet dissolved by methanol. After drying membranes, the migrated cells were counted in 5 high power fields per transwell unit. The mean values of each sample were determined by triplicate assays.

### 3C-qPCR

In summary, a total of 10 million cells from various pancreatic cancer cell lines (PANC-1 and Capan-1) were harvested and subjected to crosslinking and lysis procedures. Genomic DNA was then digested using the Hind III restriction enzyme. For the 3C assay, primers were carefully designed to be located within 50 base pairs upstream of the Hind III restriction sites at PVT1 or MYC promoter, or a negative control region, to measure the interaction frequency between PVT1 and MYC promoter. The resulting 3C ligation products were subsequently quantified through SYBR Green-based PCR. The sequences of the primers used in this study can be found in the **Supplementary Materials**.

### CUT&RUN

The CUT&RUN Assay Kit from Vazyme (catalog number HD101) was utilized for this procedure. To summarize, a collection of 100,000 viable cells were gathered and secured onto Concanavalin A magnetic beads, which facilitated subsequent buffer and reagent interchanges. The cells’ outer membranes were made permeable with digitonin, thereby allowing the primary antibody and the pG-MNase fusion enzyme to penetrate the nuclei. Upon the introduction of calcium ions, the pG-MNase was activated, meticulously severing the targeted chromatin fragments. This process allowed the fragments to disengage from the genomic chromatin, migrate outside the cell, and ultimately be collected in the supernatant. The DNA was then refined through the use of DNA extract beads. The levels of binding activity of MYC were scrutinized via quantitative PCR (qPCR) and were exhibited in terms of percent input. The fold enrichment was determined using the ΔΔCT method. The sequences of the primers utilized in this analysis are detailed in the **Supplementary Materials**.

### CRISPR activation and interference (CRISPRa/i)

The short guide RNAs (sgRNAs) utilized in CRISPR interference and activation (CRISPRi/a) were crafted with the aid of the CRISPOR tool, accessible at http://crispor.tefor.net/. The sgRNA sequences, detailed in the upplementary materials, were intended to target the PVT1 or MYC promoter. These sgRNAs were synthesized via *in vitro* transcription method by BeyoCRISPR™ One-Step sgRNA Synthesis Kit (Beyotime, catalog number D7081S). For the transfection stage, pancreatic cancer cells that stably overexpressed the dCas9-KRAB or dCas9-VP64 fusion protein received the sgRNAs. The transfection was facilitated by Lipofectamine™ 3000 reagent (Invitrogen, catalog number L3000015). After a period of 48 hours following transfection, the cells were collected. The expression levels of PVT1 or MYC were then evaluated employing both reverse transcription quantitative PCR (RT-qPCR).

## Results

### Differential gene expression analysis and functional enrichment analysis of PVT1-MYC duet-related genes

**Figure 1:**
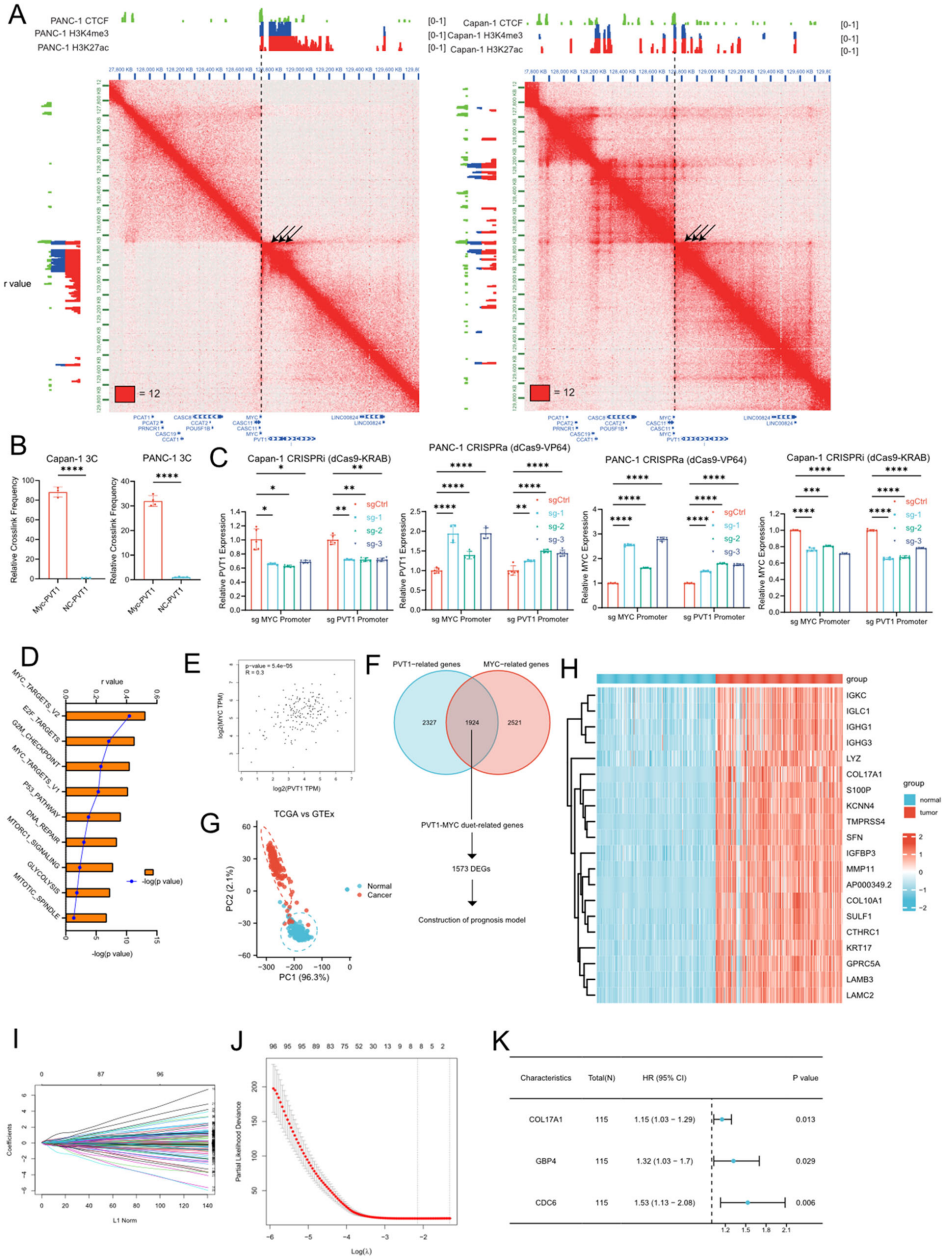
Identification of PVT1-MYC duet-related DEGs and construction of the PVT1-MYC duet-related prognostic signature. **(A)** Hi-C and ChIP-seq data of PANC-1 and Capan-1 around chr8q24 **(B)** 3C-qPCR of PVT1 and MYC promoters in PANC-1 or Capan-1 cells. T-test, ****P < 0.0001 **(C)** CRISPRa or CRSPRi targeting PVT1 or MYC promoter. Two-way ANOVA, *P < 0.05, **P < 0.01, ***P < 0.001, ****P < 0.0001. **(D)** Correlation analysis between PVT1 expression in TCGA and enrichment scores of “hallmarks” datasets in MSigDb. **(E)** Correlation analysis between PVT1 and MYC expression in TCGA pancreatic cancer samples. **(F)** Venn diagram of PVT1-MYC duet-related genes and flowchart of downstream analysis (DEGs, differential expressed genes). **(G)** PCA based on PVT1-MYC duet-related genes of tumor and normal samples of the TCGA and GTEx datasets. **(H)** Heatmap of PVT1-MYC duet-related DEGs between normal (GTEx) and tumor (TCGA) samples. **(I)** LASSO coefficient profiles of prognostic PVT1-MYC duet-related genes s. **(J)** The most proper log (λ) value in LASSO regression analysis. **(K)** The results of multivariate Cox regression analysis for 3 significantly PVT1-MYC duet-related genes contributing to OS in PC.

To gain the insight of PVT1-MYC duet biological meaning in PDAC, we first analyzed the Hi-C and ChIP-seq of H3K4me3 (promoter), H3K27ac (active promoter and enhancer), and CTCF (chromatin structural protein) from our previous work (17), to find the chromatin interaction around the promoter of PVT1 (**Figure 1A**). The Hi-C data of PANC-1 (derived from primary PDAC) and Capan-1 (derived from liver metastasis of PDAC) that there were strong interactions between the promoters of PVT1 and MYC, and these promoters had H3K27ac modification, suggesting that the promoter of PVT1 could act as the enhancer of MYC and vice versa, which cause co-expression of PVT1 and MYC and further promote PDAC progression via MYC related pathways. Furthermore, MYC promoter interacted with the promoter and gene body of PVT1 more and formed the “stripe” (26), which cause stronger chromatin interaction in PANC-1. Our previous work showed that PVT1 expression was higher in PANC-1 than Capan-1 (12), which was consistent with our Hi-C and ChIP-seq data. We subsequently confirmed the interaction between PVT1 and the MYC promoter in PANC-1 and Capan-1 cell lines through 3C-qPCR experiments (**Figure 1B**). Furthermore, we demonstrated that this interaction enhances the expression of both genes through CRISPR interference (CRISPRi) and CRISPR activation (CRISPRa) experiments (**Figure 1C**). Meanwhile, we performed ssGSEA to calculate enrichment score of gene sets of MSigDb database in TCGA PDAC tissues. Correlation analysis showed that PVT1 was associated with several pathways related to cancer progression, and MYC targets showed the strongest positive association (**Figure 1D**). Consistently, correlation analysis of PVT1 and MYC expression showed that PVT1 expression was significantly positively correlated to MYC expression (**Figure 1E**). These results confirmed that the “PVT1-MYC duet” played an important role in PDAC progression.

Then, we were wonder about the roles of PVT1-MYC duet in pancreatic cancer progression. We first performed correlation analysis and identified 1924 PVT1-MYC duet-related genes (as described in methods). PCA showed that the distribution of PVT1-MYC duet-related genes differs between normal pancreatic tissues and pancreatic cancer samples (**Figure 1G**). DEG analysis identified a total of 1573 DEGs were identified, and visualized by heatmap (**Figures 1F, H**). GO enrichment analysis suggested that these DEGs were mainly involved in several tumor microenvrionment-related pathways, such as cytokine-mediated signaling pathway, positive regulation of NFkB signaling, cell-substrate junction. (**Supplementary Figure 1**). Meanwhile, KEGG enrichment analysis indicated that apoptosis, TNF signaling pathway in cancer were enriched (**Supplementary Figure 1**).

### Construction of the PVT1-MYC duet-related prognostic model in pancreatic cancer

To reduce the number of genes needed for constructing the prognostic model, we first utilized LASSO regression analysis was performed on 1573 DEGs in the training cohort, which was obtained from TCGA cohort as described in methods, and 8 candidate genes were retained by the most proper value of lambda (λ) (**Figures 1I, J**). Subsequently, we established the best regression model by a stepwise multivariate Cox regression analysis and confirmed three PVT1-MYC duet-related genes significantly contributing to OS in pancreatic cancer patients (**Figure 1K**) and the risk score of each patient was calculated using the following formula: Risk score = (0.1428227×expression level of COL17A1) + (0.2784075×expression level of GBP4) + (0.4265105×expression level of CDC6). To confirm our findings, we first compared expression of 3 genes in normal pancreatic tissues, low- and high-risk pancreatic cancer samples. Consistently, the expression level of 3 genes was the highest in high-risk group, and the expression level of them was higher in low-risk group than normal pancreatic tissues (**Supplementary Figures 2B, 3B, 4B**). Similarly, higher expression of 3 genes was significantly associated with poor OS of pancreatic cancer patients (**Supplementary Figures 2A, 3A, 4A**). Then, we detected the clinicopathological correlation of 3 genes, we found that CDC6 and COL17A were associated with higher T classification (**Supplementary Figures 2F, 3F**), and CDC6 was associated with higher grade of pancreatic cancer (**Supplementary Figure 2E**).

### Evaluation and validation of the PVT1-MYC duet-related prognostic model

**Figure 2:**
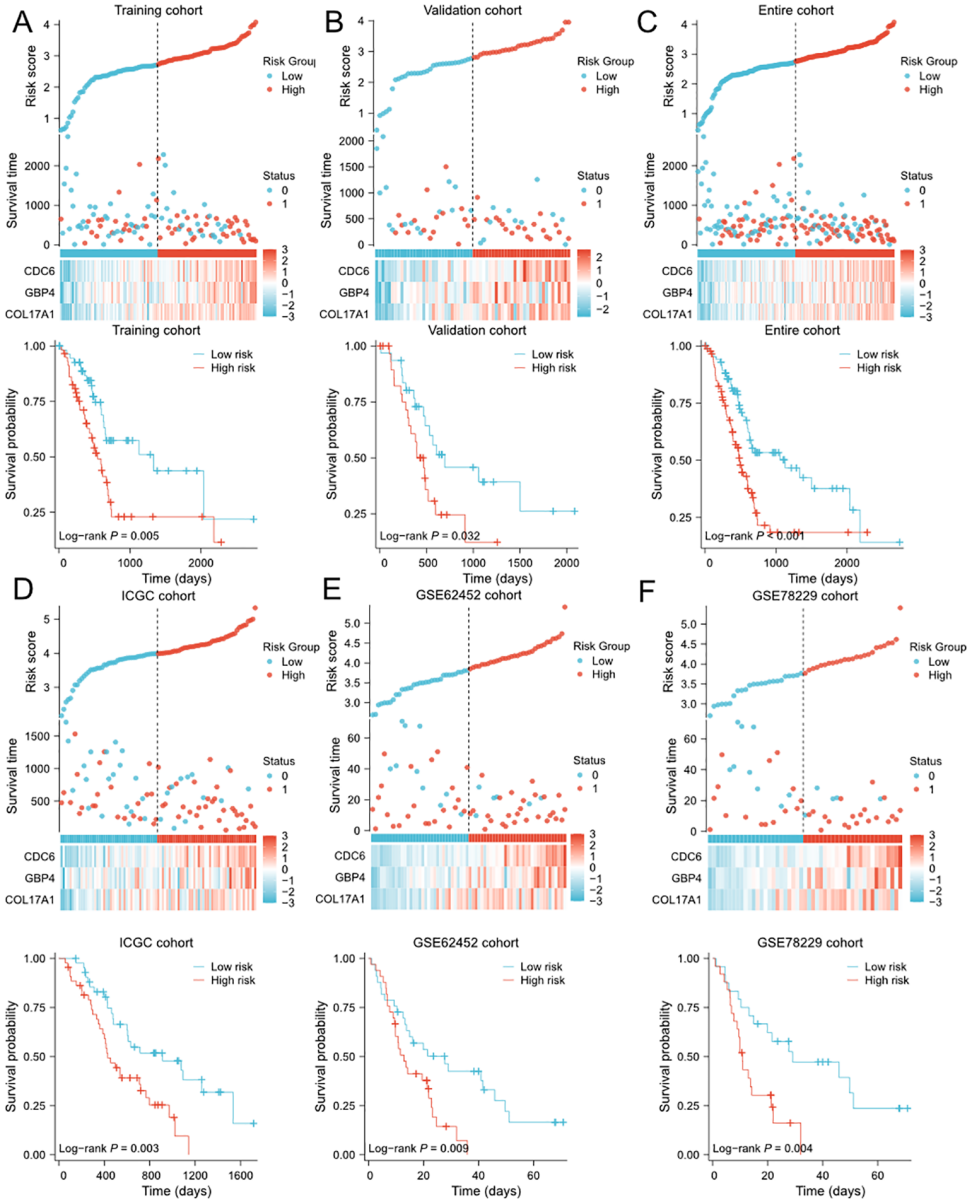
Evaluation and validation of PVT1-MYC duet-related prognostic signature in multiple cohorts **(A-F)** Distribution of risk scores, OS status overview, and heatmaps of 3 genes expression, and the Kaplan-Meier curve for OS of patients between the low- and high-risk groups in training **(A)**, validation **(B)**, TCGA **(C)**, ICGC **(D)**, GSE62452 **(E)**, and GSE78229 **(F)** cohorts.

We first evaluated prognostic model in the training cohort. Samples of training cohort was separated into the low- and high-risk groups based on the median of risk scores. The scatterplots showed that, as the expression of COL17A1/GBP4/CDC6 increased and the risk score increased, the survival time of each pancreatic cancer patient decreased and the proportion of death increased (**Figure 2A**). Meanwhile, the Kaplan–Meier survival analysis indicated that patients of all of cohorts in the high-risk group have a shorter OS than those in the low-risk group (**Figure 2A**). The PCA revealed that patients in low- or high-risk group were distributed into two clusters (**Supplementary Figure 5A**). To demonstrate the robustness of the prognostic signature, we performed same analyses in validation/TCGA cohorts for internal validation, and ICGC/GSE62452/GSE78229 cohorts as external validation to test the predictive efficiency. The definition of low- and high-risk groups in other 5 cohorts was same as that of training cohort and the risk score was calculated by the same formula. Consistently, patients in the high-risk group of each cohort were associated with worse prognosis than those in the low-risk group. Similarly, the expression levels of COL17A1/GBP4/CDC6 in the high-risk group were increased (**Figures 2B-F**). The PCA confirmed that patients in different subgroups could be divided into two separate directions (**Supplementary Figures 5B–F**), except for ICGC cohort. Finally, to evaluate the prognostic power of the PVT1-MYC duet risk signature, we performed the univariate and multivariate Cox regression analyses on TCGA and ICGC cohorts, combined with other clinicopathological features. According to the multivariate Cox regression analysis, the risk score was demonstrated to be an independent prognostic predictor for OS in these two cohorts (**Supplementary Tables 3, 4**).

### Establishment and validation of the predictive nomogram based on the risk signature

**Figure 3:**
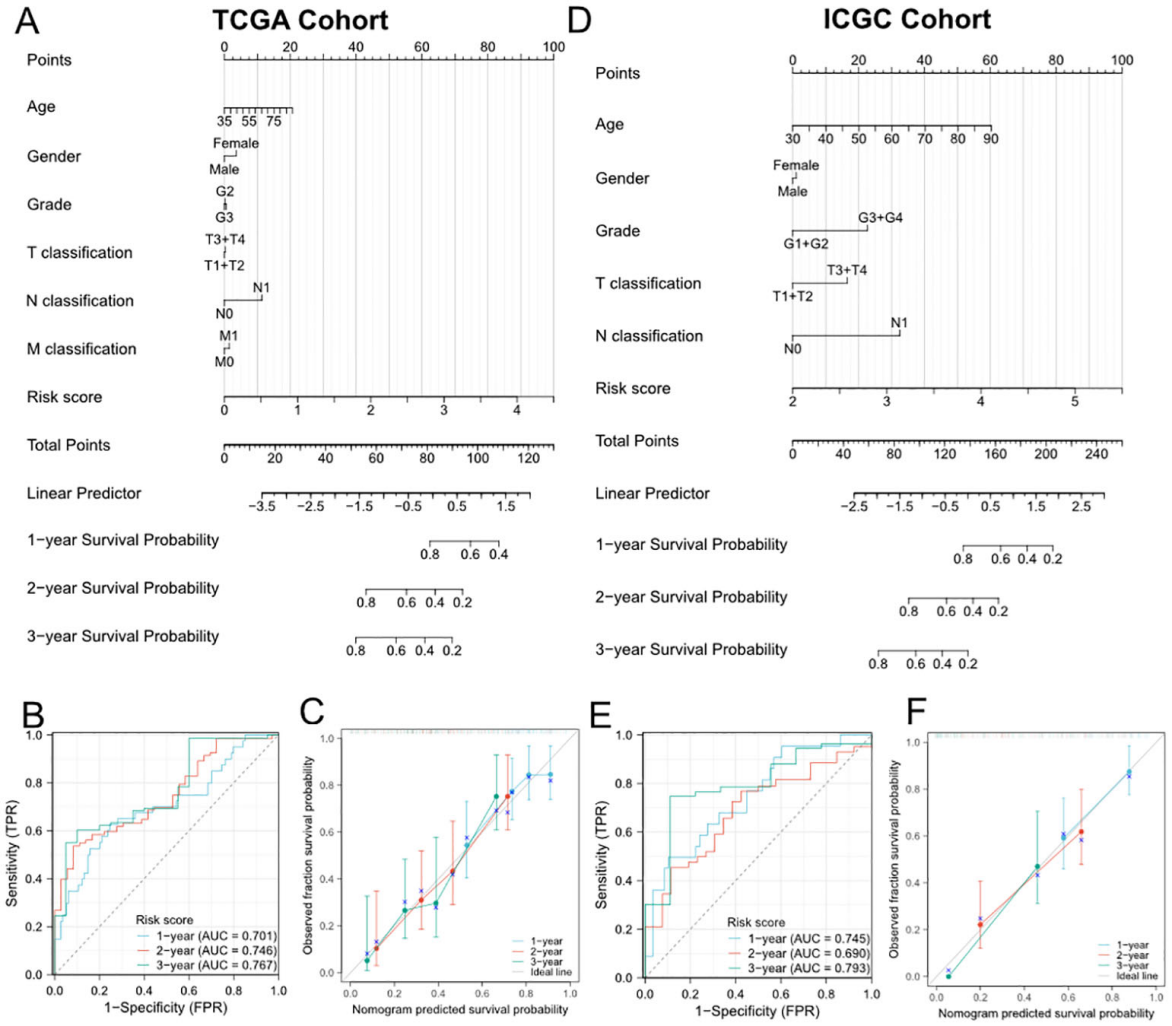
Establishment and evaluation of the predictive nomogram model. **(A, D)** Nomograms based on the risk score and clinicopathological characteristics for predicting the probability of 1-, 2-, 3-year OS in TCGA **(A)** and ICGC **(D)** cohorts. **(B)** and **(E)** Time-dependent ROC analysis of the nomogram in TCGA **(B)** and ICGC **(E)** cohorts. **(C, F)** Calibration curves of the nomogram in terms of agreement between observed and predicted 1-, 2- and 3-year survival probability in TCGA **(C)** and ICGC **(F)** cohorts.

To further improve the predictive efficiency, the risk score and other clinicopathological characteristics including age, gender, grade, and TNM stage were used to construct the predictive nomogram in TCGA and ICGC cohorts altogether. The C-index for the nomogram was 0.673 (95%CI 0.639-0.708) in TCGA cohort and 0.709 (95%CI 0.668-0.749) in ICGC cohort, indicating that the two nomograms both had well predictive performance (**Figures 3A, D**). Then, we constructed the time-dependent ROC curves and calibration curves to further evaluate the effectiveness of established nomograms. The AUCs of ROC curves for predicting 1-, 2-, and 3-year survival were 0.701, 0.746 and 0.767 in TCGA cohort (**Figure 3B**), 0.745, 0.690 and 0.793 in ICGC cohort (**Figure 3E**). Besides, the calibration curves presented satisfied coherence between observed and predicted 1-year, 2-year and 3-year OS in both cohorts (**Figures 3C, F**).

### Functional enrichment analyses of DEGs and somatic mutation profiles between high- and low-risk groups

**Figure 4:**
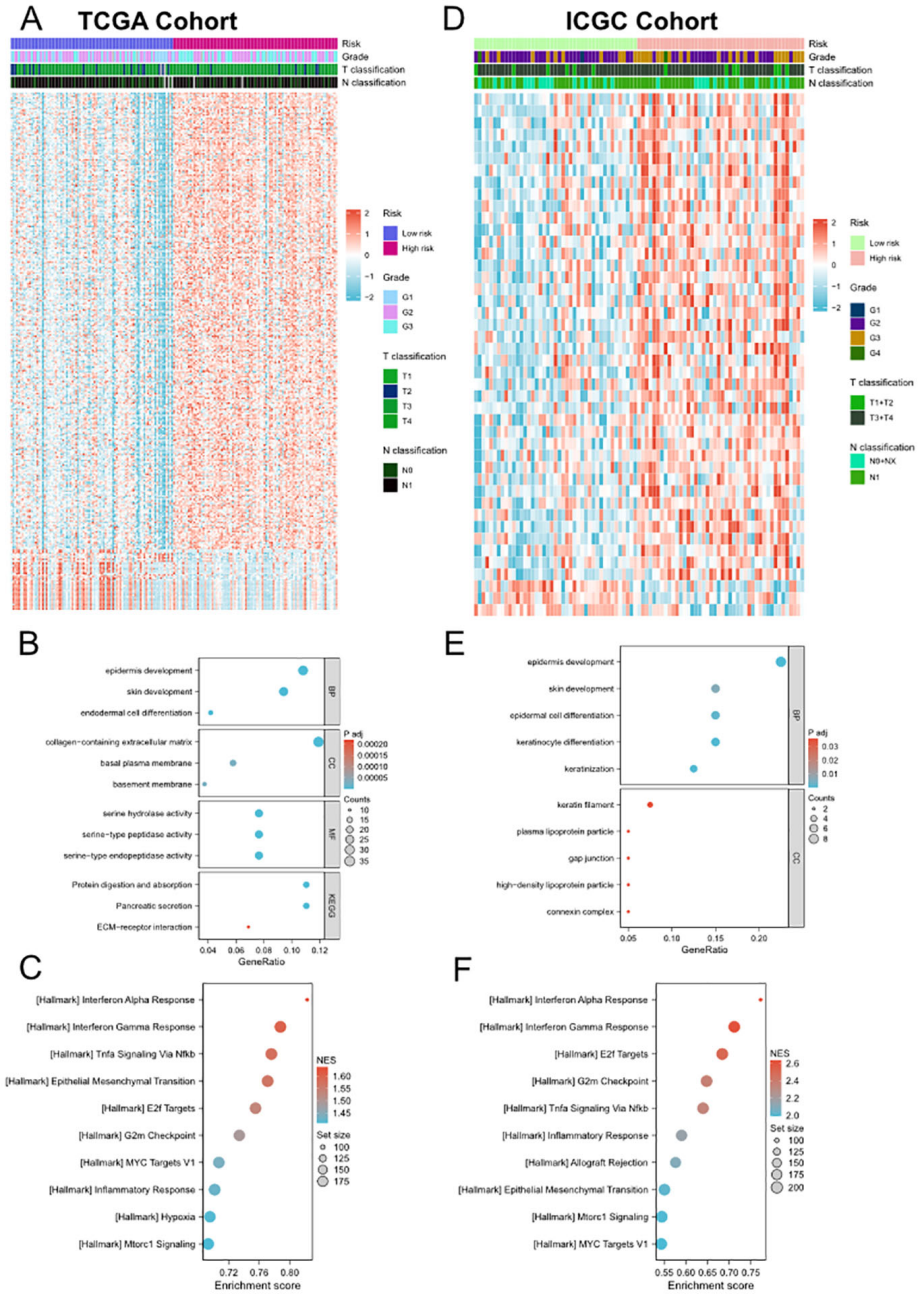
Differential gene expression analysis, GO and KEGG enrichment analyses and GSEA between low- and high-risk groups. **(A, D)** Heatmap of the DEGs between the low- and high-risk groups in TCGA **(A)** and ICGC **(D)** cohorts. **(B)** and **(E)** Representative terms of GO and KEGG enrichment analyses between the low- and high-risk groups in TCGA **(B)** and ICGC **(E)** cohorts. **(C, F)** Representative hallmarks of GSEA between the low- and high-risk groups in TCGA **(C)** and ICGC **(F)** cohorts.

In order to further explore the biological functions and pathways associated with the risk signature, we first performed DEG analysis between the high-risk and low-risk groups in TCGA and ICGC cohorts and visualized them in heatmaps (**Figures 4A, D**) and volcano plots (**Supplementary Figures 6A, B**). A total of 334 DEGs were identified in TCGA cohort, including 295 upregulated and 39 downregulated genes. Moreover, in ICGC cohort, 44 DEGs were identified, including 41 upregulated and 3 downregulated genes. The GO and KEGG enrichment analysis showed that DEGs were enriched in several pancreatic function and metastasis-associated terms, such as pancreatic secretion, epidermal cell differentiation, ECM-receptor interaction, collagen-containing extracellular matrix (**Figures 4B, E**). Subsequently, we performed GSEA to further identify PVT1-MYC duet risk signature associated pathways. The results of GSEA demonstrated that DEGs mainly enriched in several pathways associated with immune response, cancer proliferation and metastasis, such as Interferon alpha/gamma response, TNFα signaling, MYC/E2F targets, and epithelial mesenchymal transition (**Figures 4C, F**).

**Figure 5:**
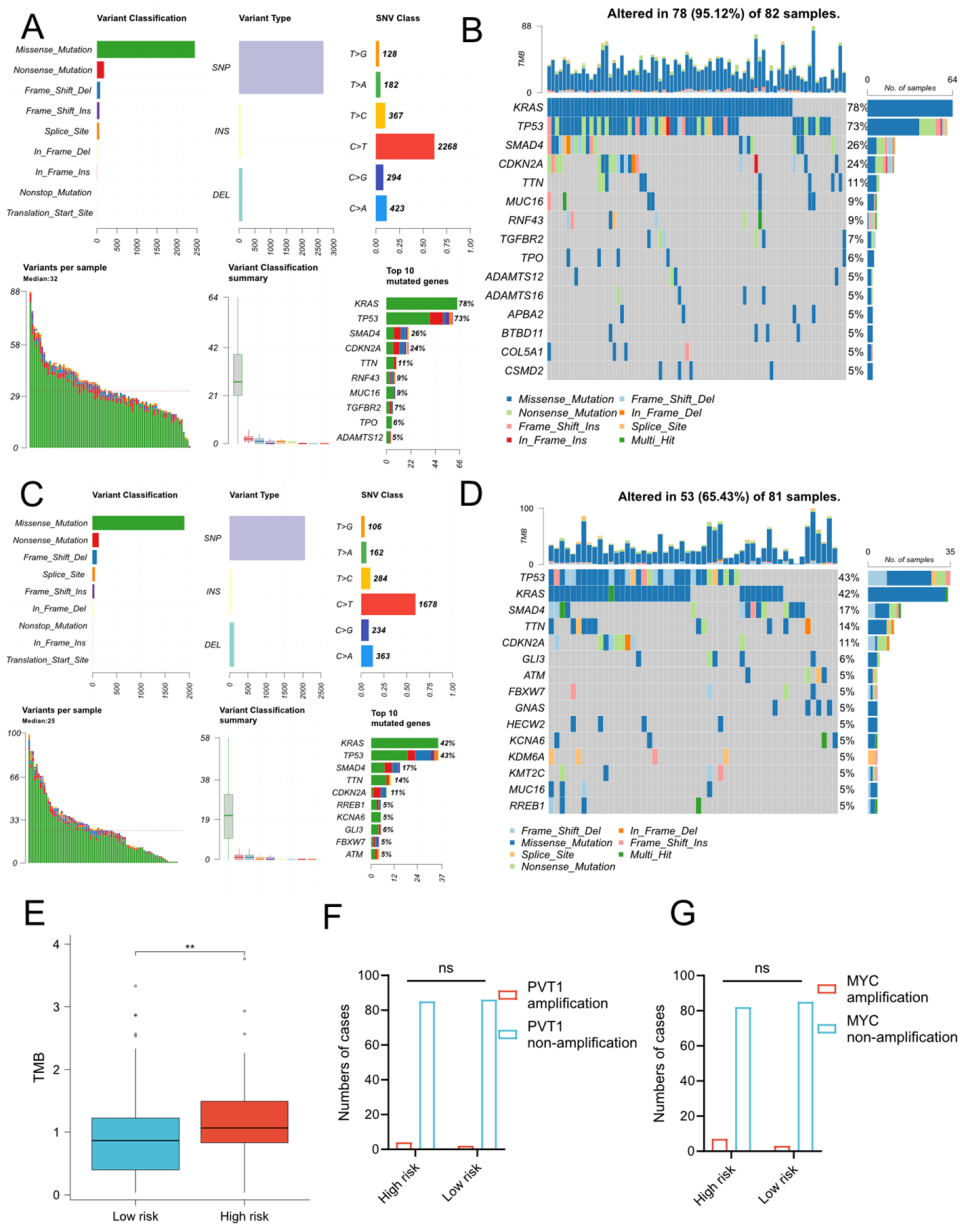
Somatic mutation profiles between low- and high-risk groups in TCGA cohort. **(A-D)** MAF-summary plots and waterfall charts of somatic mutations in the high-risk group **(A, B)** and low-risk group **(C, D)**. **(E)** Comparison of TMB between two risk groups. The unit of TMB is mutations/MB. T-test, **P < 0.01. **(F–G)** Comparison of amplification status of PVT1 **(F)** and MYC **(G)** between high and low-risk groups. ns, No significance.

To explore whether the risk signature correlated to the mutational landscapes of pancreatic cancer patients, we compared the somatic mutation profiles between the low- and high- groups in TCGA cohort (**Figures 5A–D**). Notably, the mutation frequency in the high-risk group was 95.12%, while 65.43% in the low-risk group, indicating that the mutation frequency increased along with the risk signature. Moreover, KRAS and TP53 were the top two genes with the highest mutation frequencies in both subgroups, but KRAS mutation frequency of high-risk group was much higher than low-risk group, and TP53 mutation frequency higher than KRAS mutation frequency in low-risk group. Meanwhile, we compared TMB between low- and high-risk group and we found that patients with higher risk scores demonstrated significantly higher TMB levels (**Figure 5E**). However, there is no difference of PVT1/MYC amplification between high- and low-risk groups (**Figures 5F, G**). We further conducted the same analyses in ICGC cohort and found similar results (**Supplementary Figure 7**). However, there was no difference in the amplification status of PVT1 and MYC between the high-risk and low-risk groups.

### Correlation between risk signature and immune features

**Figure 6:**
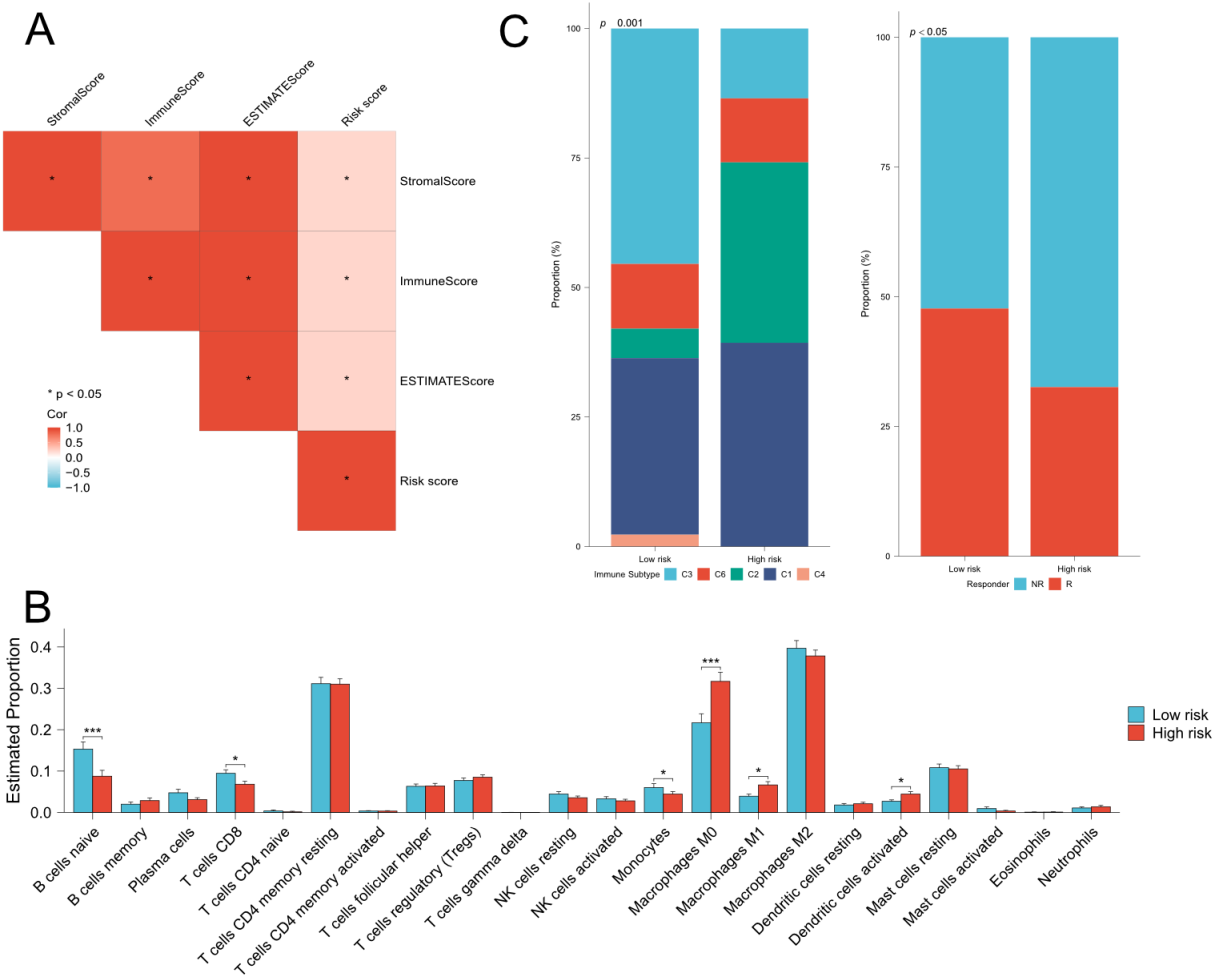
Estimation of immune cell infiltration and prediction of ICB responses in TCGA cohort. **(A)** Correlation analysis among risk score, stromal score,immune score and estimate score. **(B)** Comparison of 22 types immune cells between low- and high-risk groups. **(C)** (left) Comparison of immunesubtype proportion between low- and high-risk groups. C1: Wound-healing, C2: IFN-gamma dominant, C3: Inflammatory, C4: lymphocyte depleted,C6: TGF-beta dominant. (right) Comparison of the proportion of responder of immunotherapy between low- and high-risk groups. Fisher’s exact test,Q28 *P < 0.05, **P < 0.01, ***P < 0.001, ****P < 0.0001.

Highly heterogenous tumor microenvironment made pancreatic cancer poor prognosis. To further investigate the relationship between risk signature and immune cell infiltration, we first performed ESTIMATE analysis on TCGA cohort and ICGC cohort. ESTIMATE analysis showed that risk score positively correlated to estimate score, stromal score and immune score (**Figure 6A**). Then we used CIBERSORT algorithm to obtain the composition and correlation of each type of tumor-infiltrating immune cells. The results showed that the risk signature was negatively associated with CD8^+^ T cells (**Figure 6B**, **Supplementary Figure 8B**). Subsequently, we analyzed the correlation between risk signature and immune subtype. The classification of immune subtypes showed that five subtypes were identified in TCGA cohort and ICGC cohort (**Figure 6C**; **Supplementary Figures 8E, F**). The proportion of C1 (wound healing) or C2 (IFN-γ dominant) subtypes was significantly higher and the proportion of C3 (inflammatory) subtype was significantly lower in high-risk group, which suggested an unfavorable prognosis of high-risk pancreatic cancer patients. Furthermore, we predicted the immune checkpoint blockade (ICB) therapeutic responses on TCGA and ICGC cohort and compared the ICB response rate between low- and high-risk groups. We found that the response rates were significantly lower in the high-risk group in TCGA cohort and decreasing tendency was observed in ICGC cohort. These findings indicated that patients with higher risk scores might be in an immune-suppressive status.

### PVT1 expression was associated with invasion, metastasis and poor prognosis of PDAC

**Figure 7:**
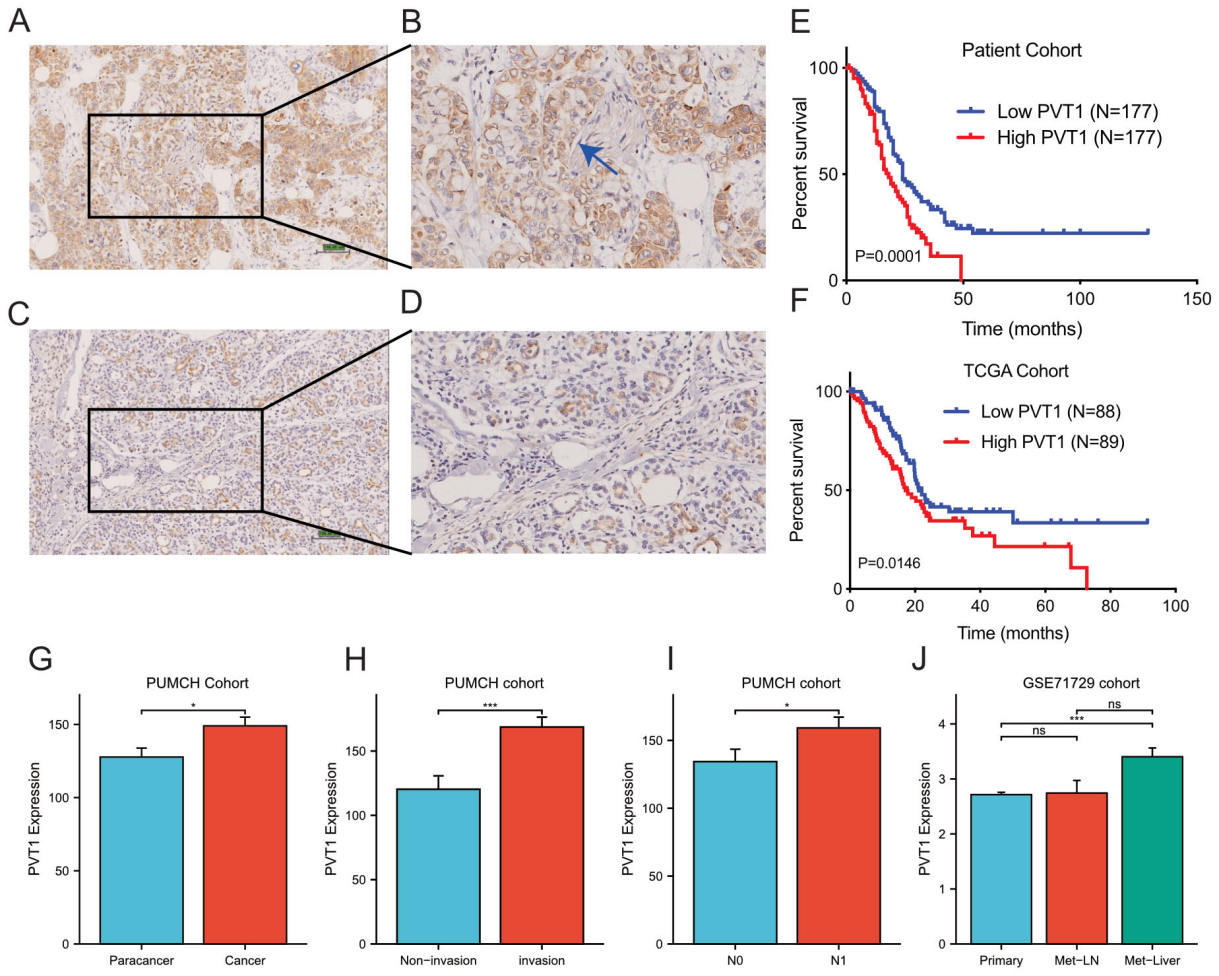
PVT1 was upregulated in PDAC tissues. **(A, B)** Representative images of PVT1 expression in PDAC tissues by ISH assays. The blue arrow in **(B)** presented the nerve in tumor tissue. **(C, D)** Representative images of PVT1 expression in adjacent non-tumor tissues by ISH assays. **(E)** Relative expression of PVT1 in PDAC tissues and adjacent non-tumor tissues. **(F)** Relative expression of PVT1 in PDAC tissues and normal pancreatic tissues from TCGA and GTEx database. **(G)** Relative expression of PVT1 in PDAC tissues or paracancer tissues in PUMCH PDAC cohort. **(H)** Relative expression of PVT1 in PDAC tissues with/without invasion in PUMCH PDAC cohort. **(I)** Relative expression of PVT1 in PDAC tissues with/without lymphatic metastasis in PUMCH PDAC cohort. **(J)** Relative expression of PVT1 in primary, lymphatic metastasis, liver metastasis of PDAC tissues in GSE71729 dataset. ns, no significance. * p < 0.05; *** p < 0.001.

The above findings were based on database and bioinformatics. Therefore, to validate these results, we used a total of 344 FFPE PDAC tissues with 298 paired paracancer tissues to detect the relative expression of PVT1 by ISH assays (**Figures 7A–D**). We found that PVT1 was specifically expressed in PDAC cells but not the stroma (**Figures 7A, B**). PVT1 expressed relatively less in ductal cells of paracancer tissues (**Figures 7C, D**). The data showed that the PVT1 expression level was significantly elevated in PDAC tissues compared with paracancer tissues (Mann-Whitney U test, P=0.0201, **Figure 7G**). To validate our findings, we analyzed the PVT1 expression data from the external database. We found that PVT1 expression was significantly higher in PDAC tissues from TCGA (Clinicopathological parameters were listed in **Supplementary Table 2**) than normal pancreas from GTEx (**Supplementary Figure 9A**). Datasets from Oncomine also showed that higher PVT1 expression in PDAC tissues compared with paracancer tissues (27–29) (**Supplementary Figures 9B–D**). These results demonstrated that PVT1 was upregulated in PDAC, indicating PVT1 may play an important role in PDAC progression.

As function and clinical relevance of MYC had been widely demonstrated, we next focused on the validation of PVT1 and signature genes. To explore the clinical relevance of PVT1, we first compared PVT1 expression in subgroups of PUMCH PDAC cohorts (**Supplementary Table 2**). We found that PVT1 expression significantly correlated to invasion and N1 classification (**Supplementary Table 2**; **Figures 7H, I**). Moreover, GSE71729 datasets including gene expression data of primary and metastatic PDAC. We found that PVT1 expression was significantly higher in liver metastasis, compared with primary cancer and regional lymph node metastasis (**Figure 7J**). Together, these results demonstrated that PVT1 was associated with invasion and metastasis of PDAC.

Then we utilized Kaplan-Meier survival analysis to determine the impact of PVT1 on prognosis. The 344 PDAC patients were divided into two balanced groups: high expression (H-score ≥146.25, n=172) and low expression (H-score <146.25, n=172). Subsequently, Kaplan-Meier and log-rank test were performed to investigate the relationship between PVT1 expression and patients’ overall survival (OS). We observed that PVT1 expression level was negatively associated with overall survival of PDAC patients (**Figure 7E**). Meanwhile, to consolidate our findings, we analyzed RNA-seq data of 178 PDAC patients from TCGA database. Same as before, Kaplan-Meier analysis showed that the higher PVT1 expression significantly correlated to poor prognosis of PDAC (**Figure 7F**). Taken together, PVT1 expression level can indicate the prognosis of PDAC patients.

Finally, we conducted univariate and multivariate analysis to assess the prognostic value of PVT1 of PDAC patients (**Supplementary Table 5**). Univariate analysis showed that differentiation, local invasion, N classification and PVT1 expression were significantly related to OS of patients with PDAC. Multivariate analysis by Cox regression model confirmed that high PVT1 expression was a significant independent risk factor for PDAC patients, along with differentiation and local invasion. These Cox regression analyses were further confirmed by the TCGA cohort (**Supplementary Table 6**). Thus, PVT1 was a powerful poor prognosis predictor for most PDAC patients.

### PVT1, CDC6 and COL17A1 were associated with proliferation and migration of PDAC cells

**Figure 8:**
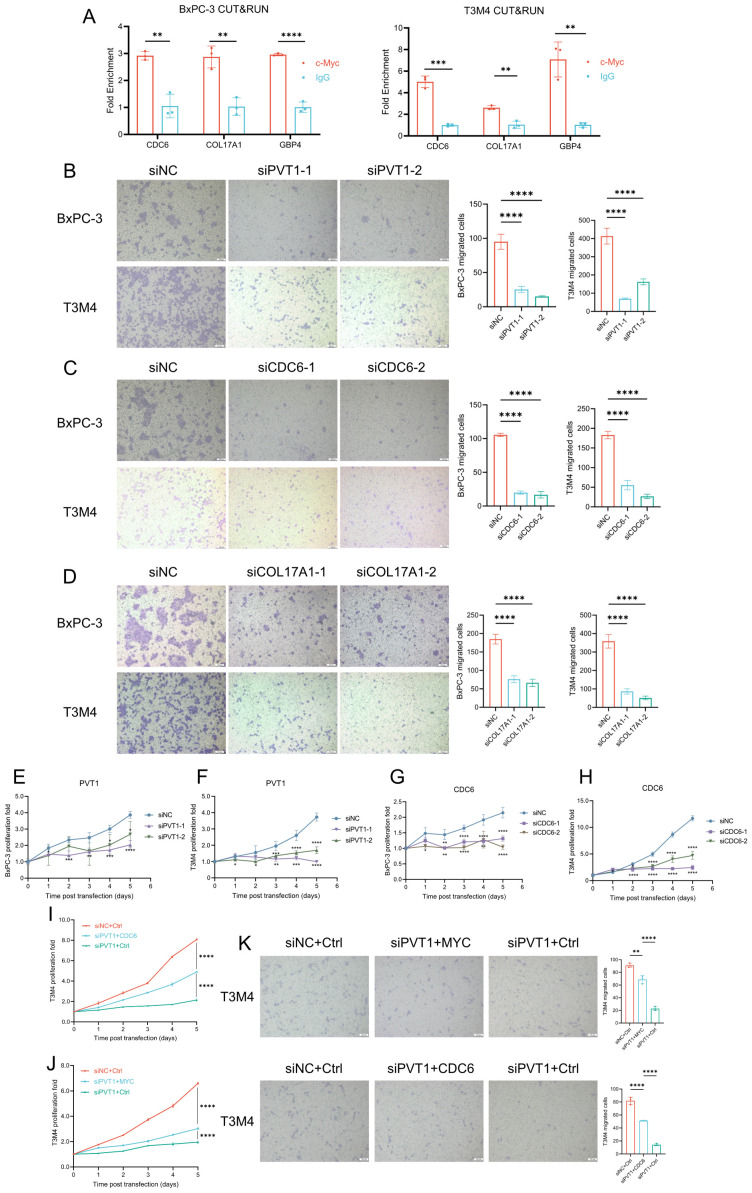
PVT1, CDC6, and COL17A1 promoted PDAC progression. **(A)** MYC CUT&RUN assay in BxPC-3 and T3M4 cell lines to test the binding activity of promoters of 3 genes. **(B-D)** Effects of silencing PVT1, CDC6, COL17A1 expression on migration of BxPC-3 and T3M4 cell lines. Scale bar: 100μm. **(E–H)** Effects of silencing PVT1 and CDC6 expression on cell proliferation of BxPC-3 and T3M4 cell lines. **(I-J)** Rescue experiments of proliferation to test whether PVT1 was dependent on CDC6 **(I)** or MYC **(J)**. **(K)** Rescue experiments of migration to test whether PVT1 was dependent on MYC or CDC6. Two-way ANOVA, *P < 0.05, **P < 0.01, ***P < 0.001, ****P < 0.0001.

As mentioned in **Supplementary Figure 3**, GBP4 is not significantly associated with clinical staging. This suggests that GBP4 is not closely related to the invasiveness and metastasis of pancreatic cancer. Therefore, we subsequently focused on exploring the functions and mechanisms of PVT1, CDC6 and COL17A1 in pancreatic cancer. We initially demonstrated through CUT&RUN assays that MYC could bind to the promoters of these three signature genes, suggesting that all three genes were downstream targets of the PVT1-MYC duet (**Figure 8A**). Then we used siRNAs to knockdown expression of genes. Transwell assays showed that knockdown of PVT1 could significantly inhibit migration of BxPC-3 and T3M4 cells (**Figure 8B**), which was consistent with the association between PVT1 expression and PDAC metastasis. Similarly, we found that knockdown of CDC6 and COL17A1 could also inhibit migration of these PDAC cells (**Figures 8C, D**). Furthermore, the proliferation assays indicated that knockdown of PVT1 and CDC6 could significantly inhibit proliferation of BxPC-3 and T3M4 (**Figures 8E-H**). We subsequently validated through rescue experiments that PVT1 can promote the proliferation and migration of pancreatic cancer cells via MYC. Moreover, since CDC6 consistently yielded positive results in previous proliferation and migration assays, it suggests that CDC6 is a key downstream factor in the promotion of pancreatic cancer progression by the PVT1-MYC duet. Similar rescue experiments demonstrated that PVT1 can also promote the proliferation and migration of pancreatic cancer cells through CDC6, further confirming that CDC6 is a crucial downstream element in the PVT1-MYC duet’s facilitation of pancreatic cancer (**Figures 8I–K**).

## Discussion

Pancreatic cancer is an extremely malignant tumor with high mortality rate, due to the complex interplay of genetic alterations, tumor microenvironment interactions, and a dense desmoplastic stroma that contributes to its resistance to therapies (30). The molecular landscape of PDAC is predominantly shaped by mutations in the KRAS oncogene, which is mutated in over 90% of cases, along with frequent mutations in TP53, CDKN2A, and SMAD4 (30). Recent advances in understanding PDAC biology have led to the identification of potential therapeutic targets, such as the Aurora kinases, cyclin-dependent kinases (CDKs), and vascular endothelial growth factor (VEGF) pathway. However, clinical responses to these targeted therapies have been limited, reflecting the heterogeneity and complexity of PDAC (31). Ongoing research aims to dissect these components to develop more effective therapies for this devastating disease. The PVT1-MYC duet, which has been implicated in the regulation of cell growth and survival in various cancers, may represent another layer of complexity in PDAC biology, potentially offering novel insights into disease progression and therapeutic intervention. Despite some basic researches of PVT1-MYC duet suggested its role in cancer progression, the clinical relevance of PVT1, the potential regulatory networks of PVT1-MYC duet and its prognostic value remained to be elucidated, especially in large patient cohorts.

Firstly, we developed a risk scoring model based on three PVT1-MYC duet-related genes (CDC6, COL17A1, GBP4) in training cohort of TCGA cohort, and further performed internal and external validation for its robustness. According to the values of hazard ratio, these 3 genes were considered as the risk genes. CDC6 was considered as the replication licensing factor, which was also associated with epithelial-mesenchymal transition inducing androgen receptor blockade therapeutic resistance in prostate cancer (32). Previous studies have indicated that CDC6 was upregulated in multiple types of cancer, including breast cancer, stomach cancer, glioma, and pancreatic cancer, which can facilitate the proliferation and invasion of cancer cells (33). COL17A1 is a cell-adhesion molecule which strengthens hemidesmosomes, and functionally contributes to tumorigenesis and progression. For instance, COL17A1 could mediate dormancy of colorectal cancer cells via FAK-YAP signaling and induce chemoresistance of colorectal cancer (34). Meanwhile, a previous study reported that COL17A1 could promote proliferation, migration, epithelial-mesenchymal transition of pancreatic cancer cells (35). GBP4 is an interferon-inducible GTPase, which plays important roles in innate immunity. Previous studies showed that GBP4 was associated with tumorigenesis and progression via modulate tumor immune microenvironment (36, 37). Besides, researches in neuroblastoma and colorectal cancer showed that Myc could positively regulate CDC6 (38) and COL17A1 (39) expression, respectively, suggesting that these two signature genes may be the downstream target of PVT1-MYC duet in pancreatic cancer.

Based on Cox regression coefficients of 3 genes, we constructed prognostic model and calculated risk score of each patient. Patients were divided into the low- and high-risk groups. Our results showed that high-risk pancreatic cancer patients had significantly poorer OS than low-risk patients, and the risk score was an independent prognostic factor, which further confirmed by ROC analysis and nomogram model. Then, we explored the difference of biological functions, mutation profiles and immune features between low- and high-risk groups in TCGA and ICGC cohorts. Functional enrichment analyses showed that several cancer progression-related terms and pathways were enriched, such as MYC/E2F targets, ECM-receptor interaction, and epithelial mesenchymal transition, consistent with the poorer prognosis of the high-risk group and suggesting that patients in the high-risk group may be at higher degree of cancer-related pathways activation. Furthermore, a much higher proportion of patients with KRAS somatic mutations were detected in the high-risk group, which also increased the risk of these patients. Similarly, high-risk patients also had significantly higher TMB. Numerous studies have demonstrated that the tumor immune microenvironment plays a pivotal role in the progression of pancreatic cancer (40–42). However, the roles of PVT1-MYC duet-related genes for pancreatic cancer immune microenvironment are still unclear. In our results, significantly lower infiltration levels of CD8+ T cells were observed in the high-risk group, suggesting that PVT1-MYC duet could induce an immunosuppressive microenvironment of pancreatic cancer. Meanwhile, our results also showed that higher risk was associated with lower ICB response rate, which was consistent with the immunosuppressive impact of PVT1-MYC duet. Furthermore, a bioinformatic study on TCGA has defined six immune subtypes: wound healing (C1), IFN-γ dominant (C2), inflammatory (C3), lymphocyte depleted (C4), immuno-logically quiet (C5), TGF-β dominant (C6). Among of them, C3 subtype had the best prognosis, while C1 and C2 subtypes were associated with less favorable outcomes (43). Our immune subtype analysis revealed that the proportion of C1 and C2 subtypes was significantly higher in high-risk groups, and the proportion of C3 subtypes was significantly lower, which was consistent with the association between immune subtypes and prognosis. ICB therapy is one of the most successful anti-cancer immunotherapies. Finally, ICB response prediction analysis showed that high-risk group had lower ICB response rate, which supported the immunosuppressive microenvironment in the high-risk group, contributing to poor prognosis of these patients.

To validate the bioinformatic findings, we performed ISH assays to detect PVT1 expression in our PDAC cohorts, including 344 PDAC tissues with 298 paired paracancer tissues, which can make the results convincing. We discovered that PVT1 was significantly upregulated in our cohort and this result was confirmed by external datasets, including TCGA, GTEx, and Oncomine. By performing the correlation analysis between PVT1 expression and clinicopathological parameters of the cohort and TCGA/GEO datasets, we found PVT1 expression was associated with invasion and metastasis of PDAC. Survival analysis revealed that PVT1 was associated with poor prognosis of PDAC, which was confirmed by TCGA cohort. These results revealed that PVT1 could be used as a prognostic indicator of PDAC, and PVT1 was critical for progression of PDAC. Furthermore, the transwell assays and proliferation assays also indicated that PVT1, CDC6 and COL17A1 were associated with PDAC proliferation and migration, which was consistent with the results of clinical analysis.

In conclusion, our study constructed a prognostic model for pancreatic cancer based on three PVT1-MYC duet-related genes to stratify patients and predict prognosis. This study also illustrated comprehensive landscape of biological function, mutation profiles, and immune features of low- and high-risk patients, supporting that this PVT1-MYC duet-related signature had potential as a novel prognostic marker. More studies are needed to reveal new perspectives about PVT1-MYC duet in pancreatic cancer progression, which may provide a new insight on pancreatic cancer therapy.

## Data Availability

The original contributions presented in the study are included in the article/**Supplementary Material**. Further inquiries can be directed to the corresponding authors.
