# Supplementary material for: Construction of a novel model based on PVT1-MYC duet-related genes for predicting survival and characterization of the tumor microenvironment in pancreatic cancer
Source: Front Immunol. 2024 Sep 23;15:1435593. doi: 10.3389/fimmu.2024.1435593 (PMC11456451; doi:10.3389/fimmu.2024.1435593)
Supplement: Supplementary file 1 [file DataSheet1.docx]

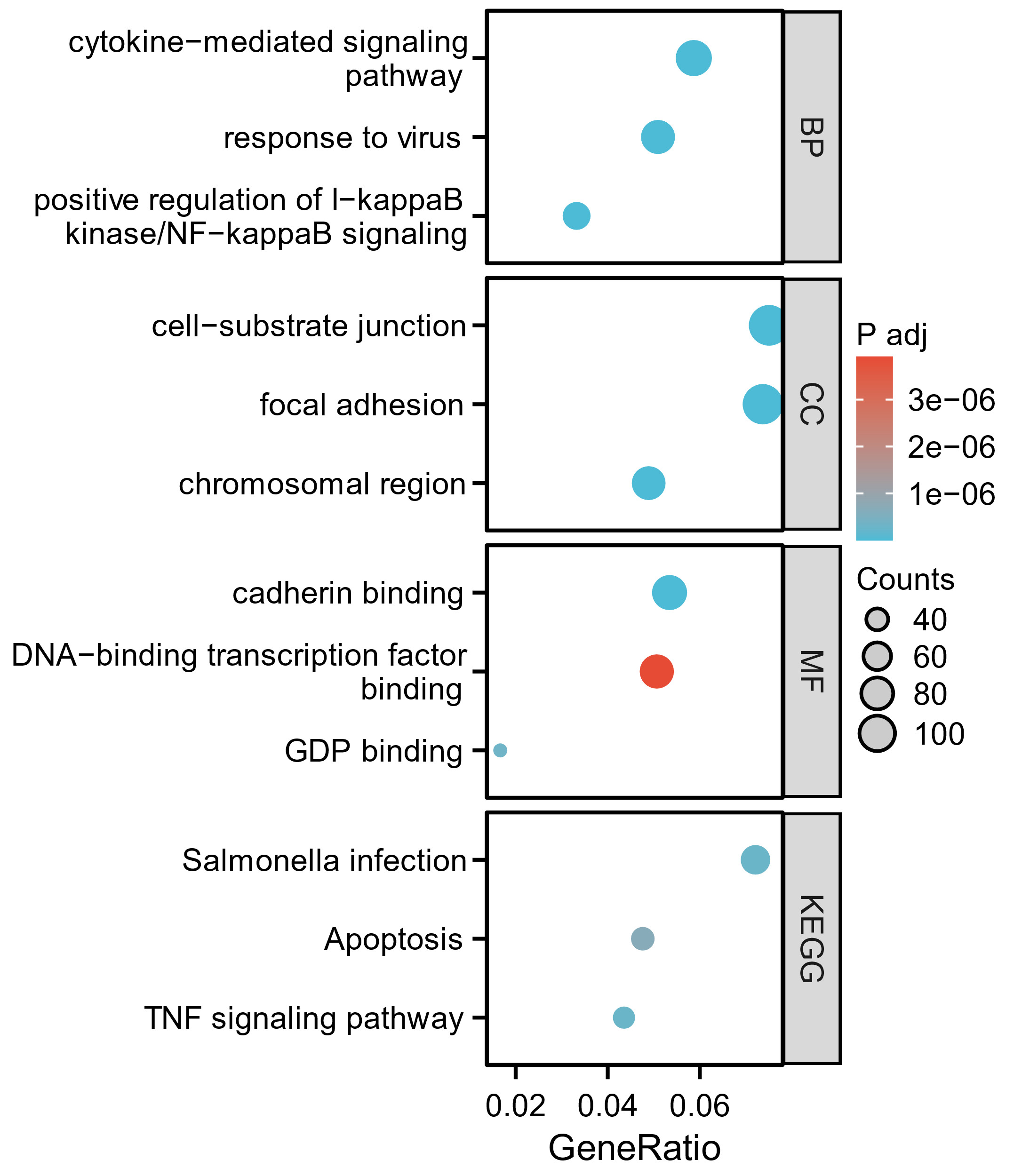


**Figure S1** GO and KEGG enrichment analyses of PVT1-MYC duet-related DEGs.


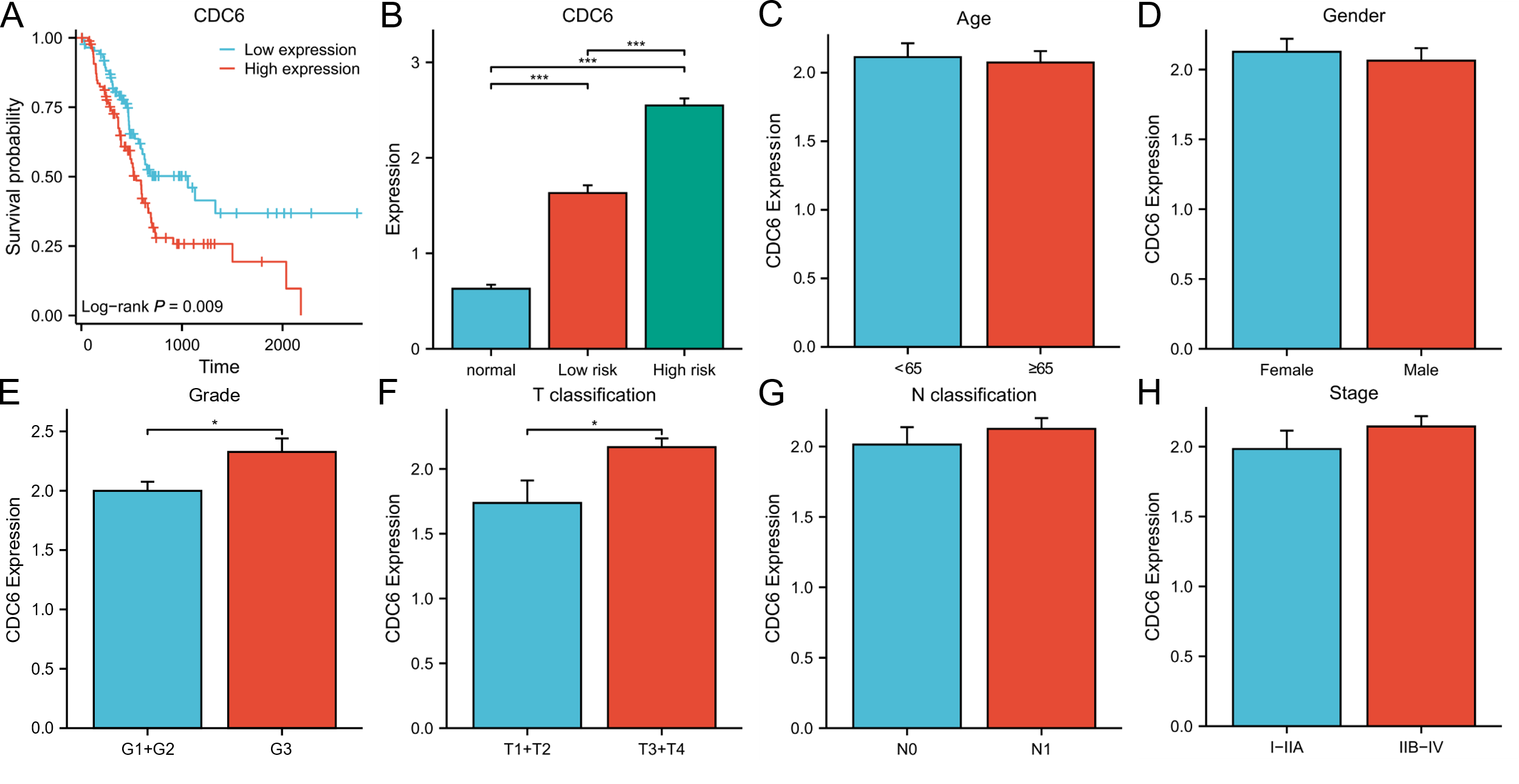


**Figure S2** Clinical relevance of CDC6 expression in TCGA cohort. (A) Kaplan-Meier curve of OS between low and high expression of CDC6. (B) Comparison of CDC6 expression among normal pancreatic samples, low-risk and high-risk pancreatic cancer samples. (C-H) Comparison of CDC6 expression between age, gender, grade, T classification, N classification, and stage subgroups.


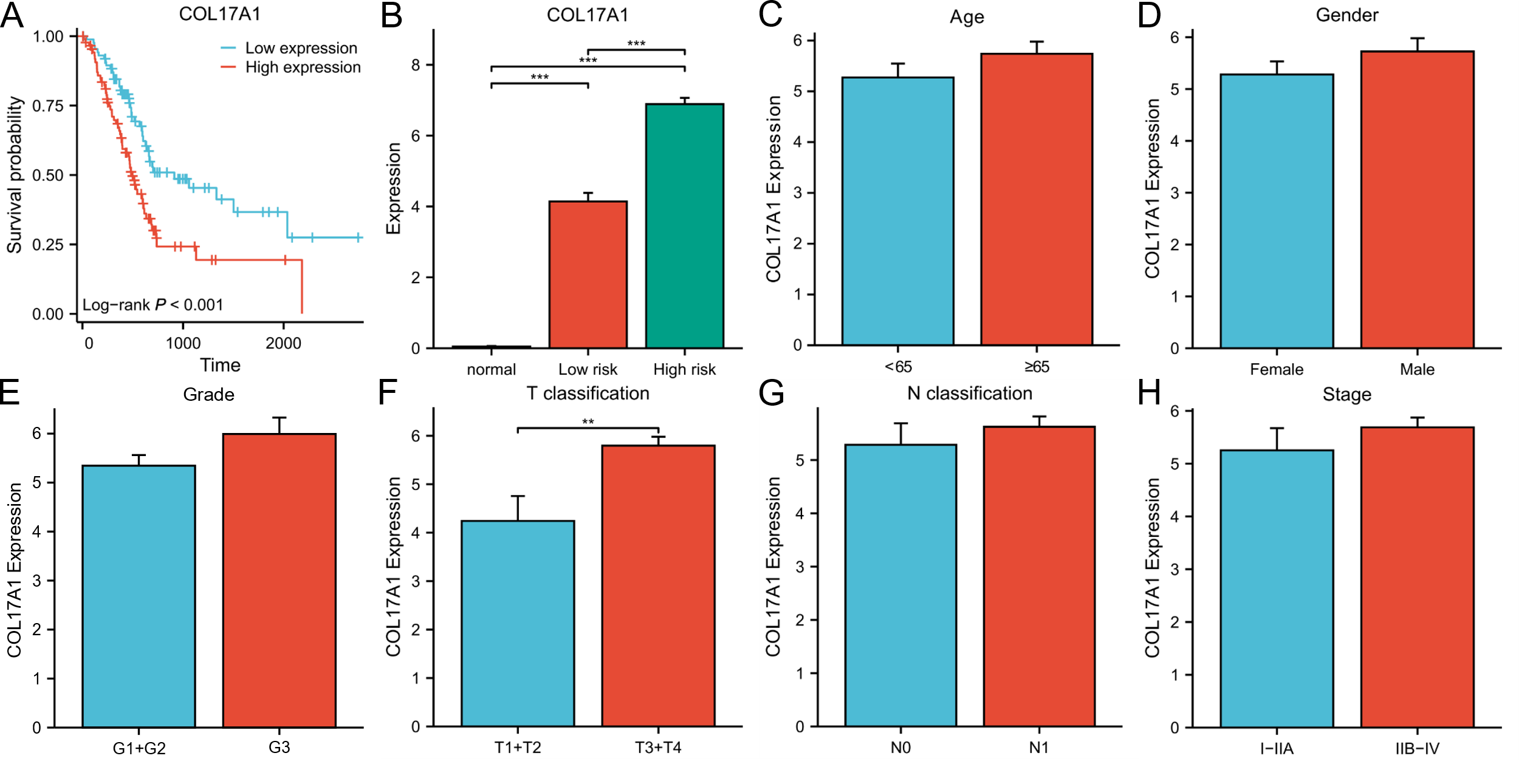


**Figure S3** Clinical relevance of COL17A1 expression in TCGA cohort. (A) Kaplan-Meier curve of OS between low and high expression of COL17A1. (B) Comparison of COL17A1 expression among normal pancreatic samples, low-risk and high-risk pancreatic cancer samples. (C-H) Comparison of COL17A1 expression between age, gender, grade, T classification, N classification, and stage subgroups.


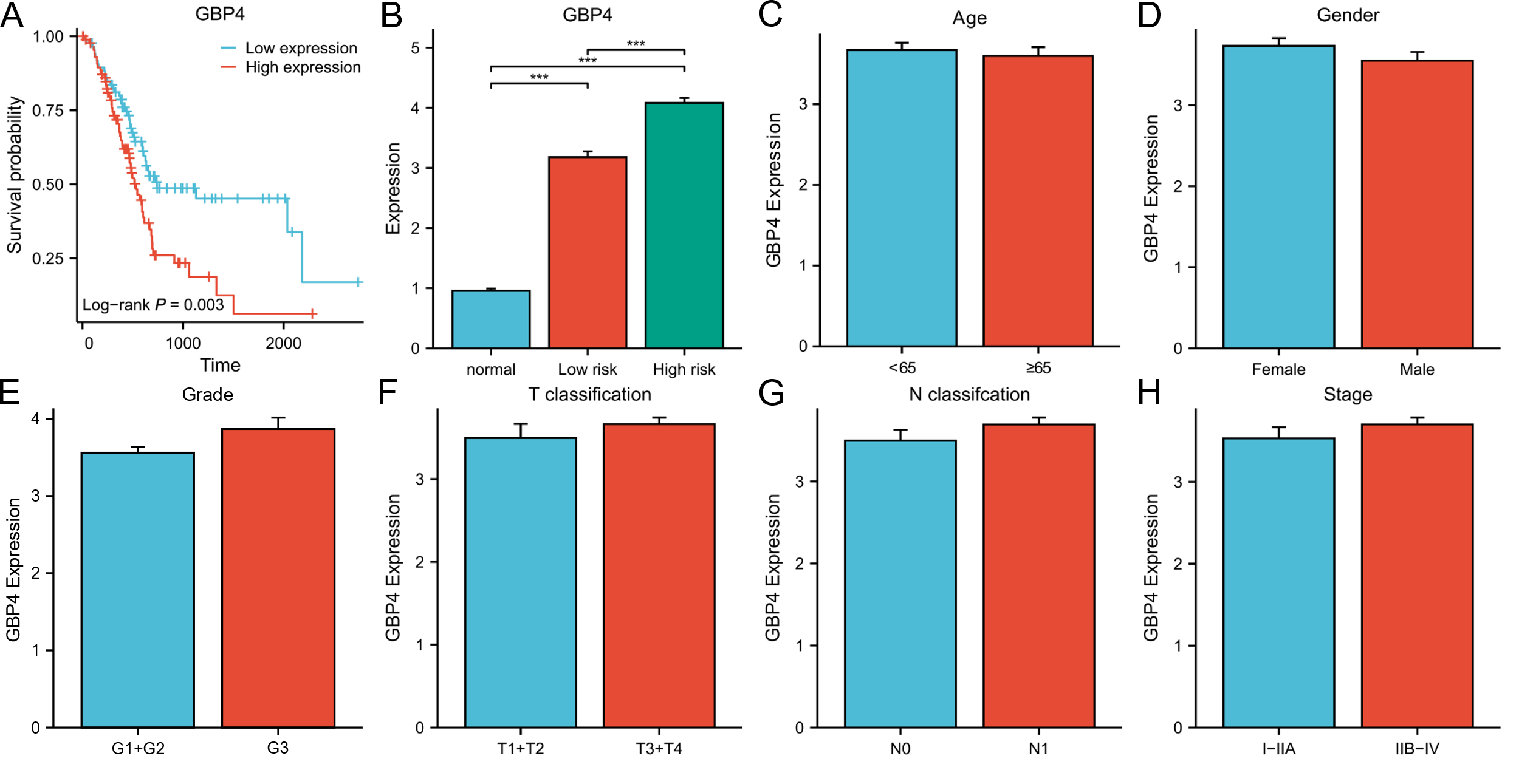


**Figure S4** Clinical relevance of GBP4 expression in TCGA cohort. (A) Kaplan-Meier curve of OS between low and high expression of GBP4. (B) Comparison of GBP4 expression among normal pancreatic samples, low-risk and high-risk pancreatic cancer samples. (C-H) Comparison of GBP4 expression between age, gender, grade, T classification, N classification, and stage subgroups.


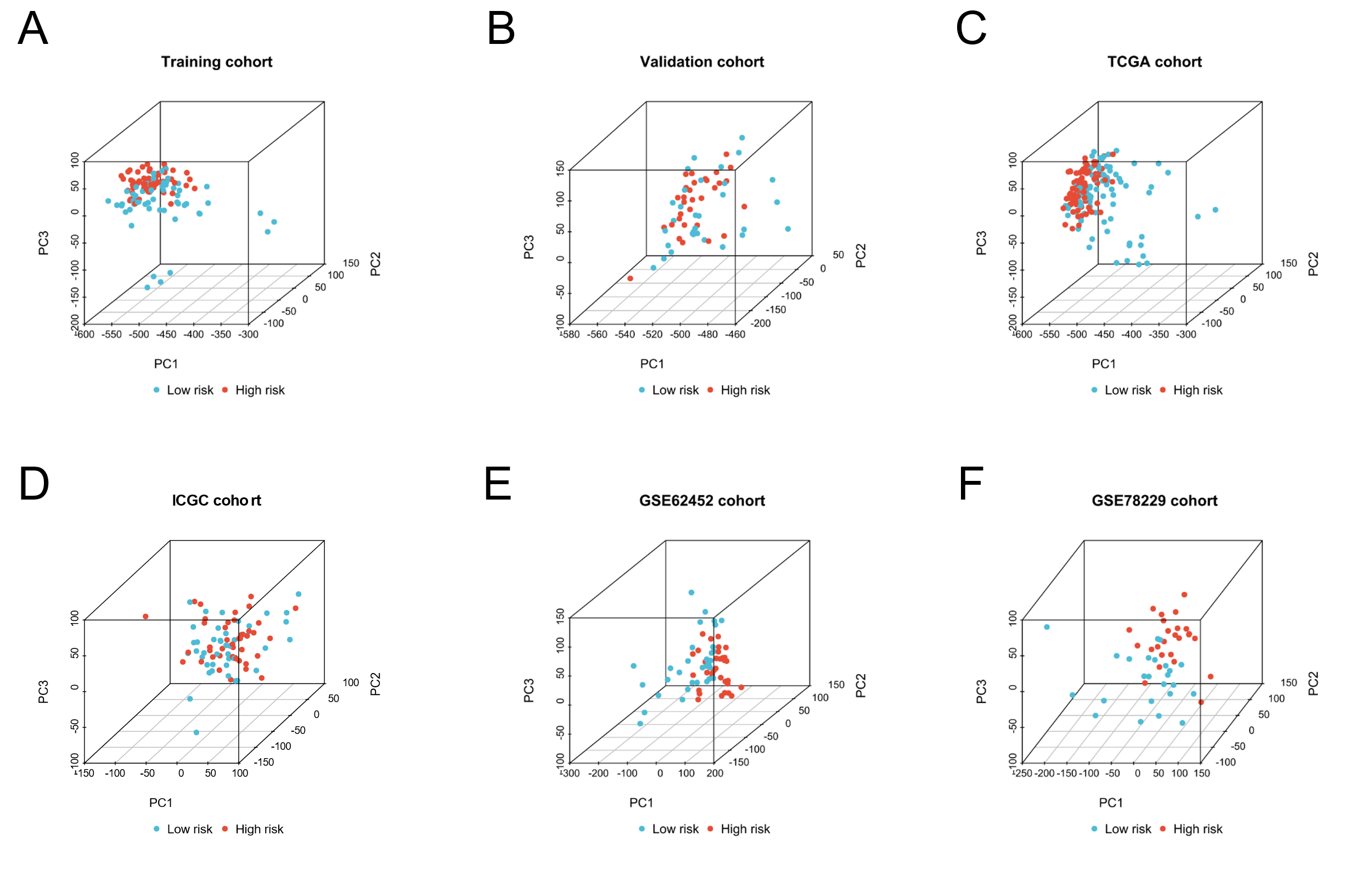


**Figure S5** PCA analysis based on the prognostic signature in training, validation, TCGA, ICGC, GSE62452, and GSE78229 cohorts.


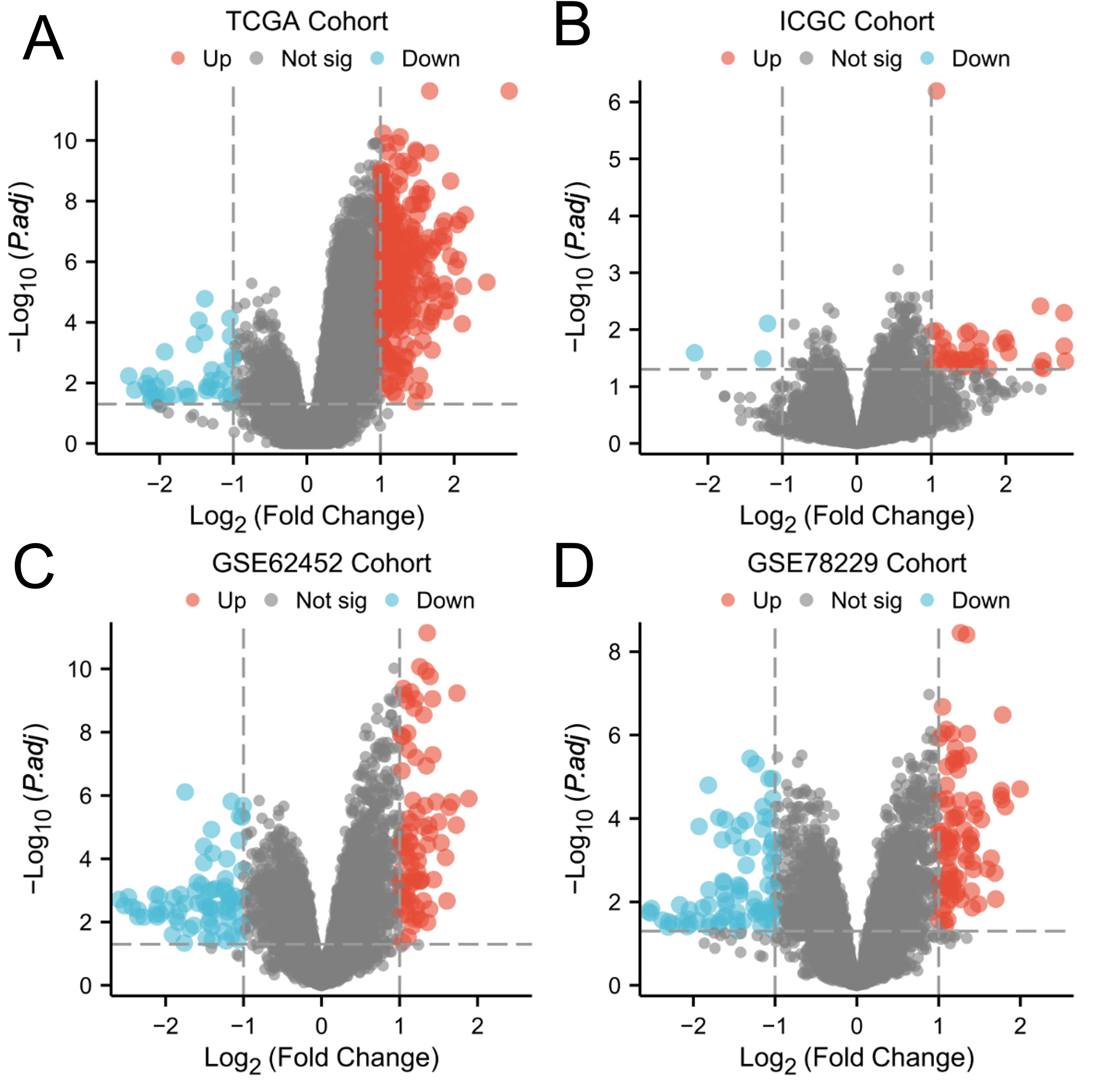


**Figure S6** Volcano plot of DEGs between low- and high-risk groups in TCGA cohort (A), ICGC cohort (B), GSE62452 cohort (C), GSE78229 cohort (D).


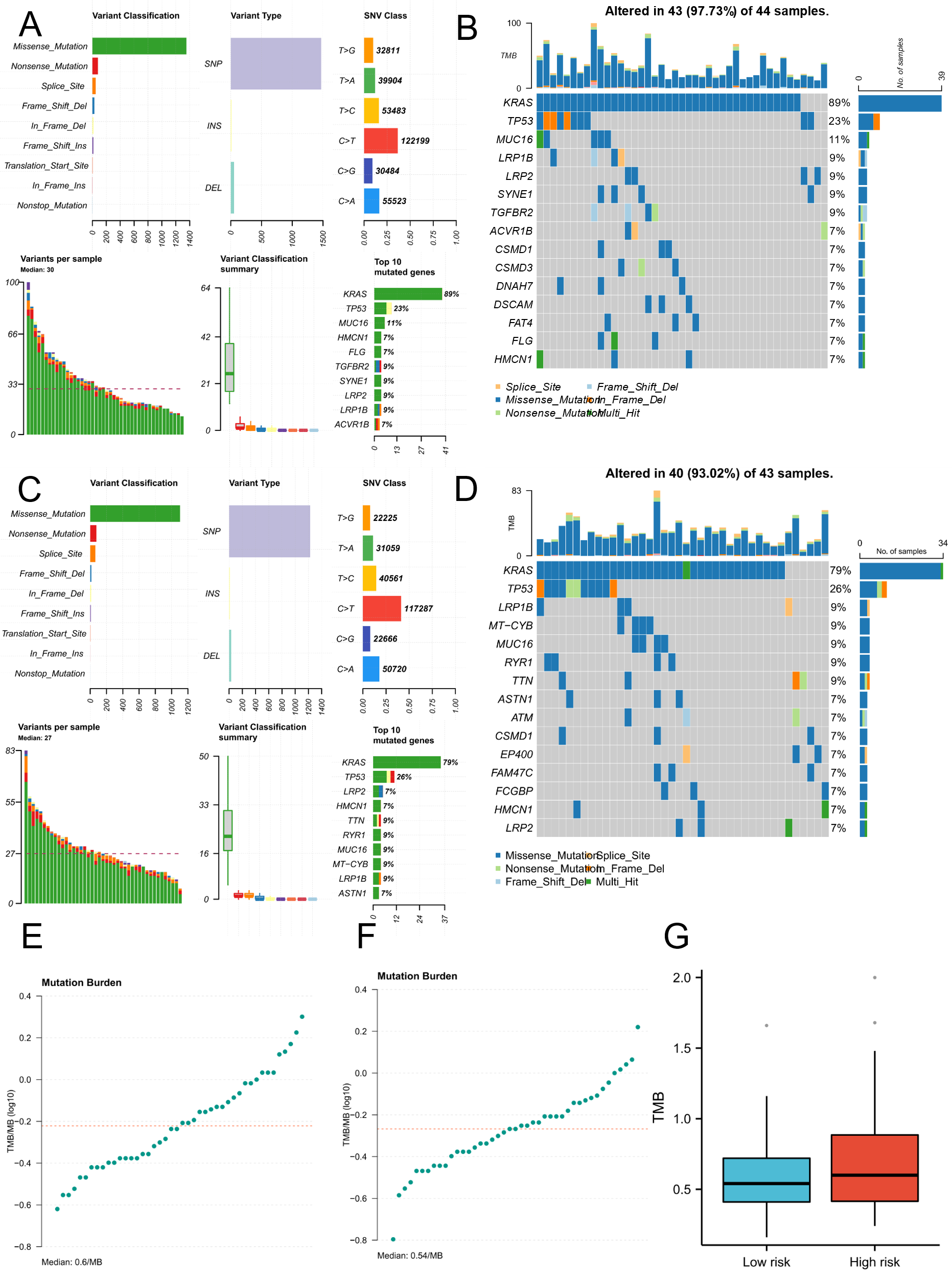


**Figure S7** Somatic mutation profiles between low- and high-risk groups in ICGC cohort. (A-D) MAF-summary plots and waterfall charts of somatic mutations in the high-risk group (A and B) and low-risk group (C and D). (E-F) Distribution of TMB in the high-risk group (E) and low-risk group (F). (G) Comparison of TMB between two risk groups.


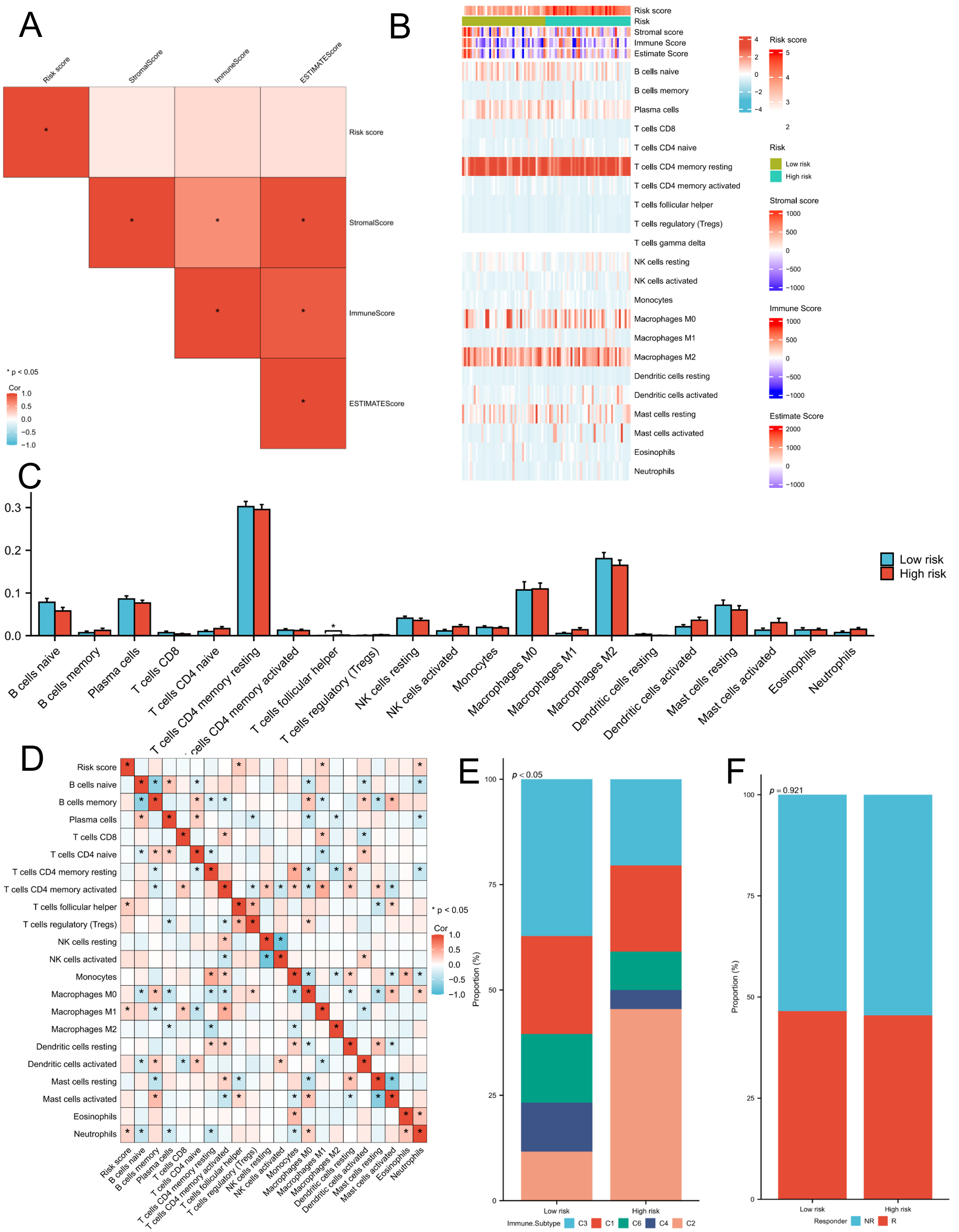


**Figure S8** Estimation of immune cell infiltration and prediction of ICB responses in ICGC cohort. (A) Correlation analysis among risk score, stromal score, immune score and estimate score. (B) Heatmap displaying the infiltrating abundances of 22 types immune cells. (C) Correlation heatmap of 22 types immune cells and the risk score. (D) Comparison of 22 types immune cells between low- and high-risk groups. (E) Comparison of immune subtype proportion between low- and high-risk groups. C1: Wound-healing, C2: IFN-gamma dominant, C3: Inflammatory, C4: lymphocyte depleted, C6: TGF-beta dominant. (F) Comparison of the proportion of responder of immunotherapy between low- and high-risk groups * P < 0.05, ** P < 0.01, *** P < 0.0001.


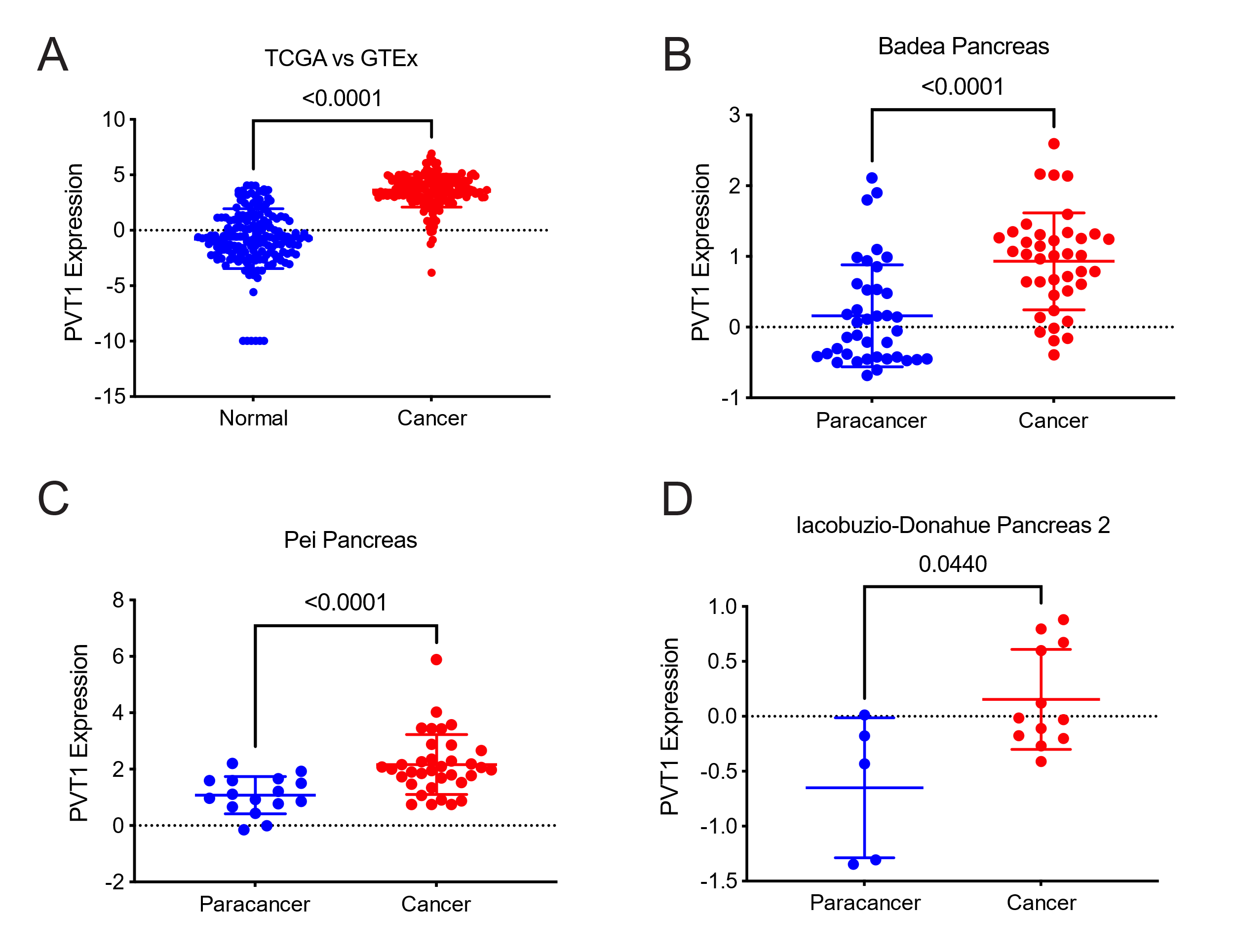


**Figure S9** PVT1 expression in TCGA, GTEx and Oncomine datasets.

**Table S1** Clinical characteristics of pancreatic cancer patients in the multiple datasets

| **Variables** | **TCGA** | **ICGC** | **GSE62452** | **GSE78229** |
| --- | --- | --- | --- | --- |
|  | N = 177 (%) | N = 87 (%) | N = 69 (%) | N = 50 (%) |
| **Age** (mean±SD) | 64.52±10.93 | 66.76±11.39 | NA | NA |
| **Gender** |  |  |  |  |
| Female | 80 (45.2) | 41 (47.1) | NA | NA |
| Male | 97 (54.8) | 46 (52.9) | NA | NA |
| **Grade** |  |  |  |  |
| G1+G2 | 125 (71.4) | 54 (62.0) | 37 (53.6) | 26 (52.0) |
| G3+G4 | 50 (28.6) | 33 (37.9) | 31 (44.9) | 23 (46.0) |
| **Stage** |  |  |  |  |
| I-II | 167 (94.4) | NA | 49 (71.0) | 49 (98.0) |
| III-IV | 7 (4.0) | NA | 19 (27.5) | 1 (2.0) |
| **T classification** |  |  |  |  |
| T1+T2 | 31 (17.5) | 13 (14.9) | NA | NA |
| T3+T4 | 146 (82.5) | 74 (85.1) | NA | NA |
| **N classification** |  |  |  |  |
| N0 | 53 (29.9) | 28 (32.2) | NA | NA |
| N1 | 124 (70.1) | 59 (67.8) | NA | NA |
| **Status** |  |  |  |  |
| Alive | 84 (47.5) | 32 (36.8) | 19 (27.9) | 14 (28.6) |
| Dead | 93 (52.5) | 55 (63.2) | 49 (72.1) | 35 (71.4) |

**Table S2** Clinical characteristics of PUMCH cohort

| **Characteristics** | **N = 344 (%)** | **P value** |
| --- | --- | --- |
| **Gender** |  | 0.0961 |
| Male | 148 (43.02) |  |
| Female | 196 (56.98) |  |
| **Age** |  | 0.0753 |
| ≤ 60 | 155 (45.06) |  |
| > 60 | 189 (54.94) |  |
| **Diabetes** |  | 0.9918 |
| No | 296 (86.05) |  |
| Yes | 48 (13.95) |  |
| **CA199** |  | 0.7352 |
| Elevated | 235 (68.31) |  |
| Normal | 55 (15.99) |  |
| **Site** |  | 0.4062 |
| Head | 206 (59.88) |  |
| Body/tail | 125 (36.34) |  |
| **Size** |  | 0.2433 |
| <3cm | 178 (51.74) |  |
| >3cm | 137 (39.83) |  |
| **Differentiation** |  | 0.9339 |
| Well | 183 (53.2) |  |
| Poor | 101 (29.36) |  |
| **Invasion** |  | **0.0005** |
| No | 110 (31.98) |  |
| Yes | 195 (56.69) |  |
| **T classification** |  | 0.7999 |
| T1 | 79 (22.97) |  |
| T2 | 176 (51.16) |  |
| T3 | 60 (17.44) |  |
| **N classification** |  | **0.0424** |
| N0 | 145 (42.15) |  |
| N1 | 185 (53.78) |  |
| **Stage** |  | 0.0724 |
| IB | 12 (3.49) |  |
| IIA | 133 (38.66) |  |
| IIB | 185 (53.78) |  |

**Table S3** Cox regression of risk score in TCGA cohort

| **Characteristics** | **Total(N)** | **Univariate analysis** | |  | **Multivariate analysis** | |
| --- | --- | --- | --- | --- | --- | --- |
|  |  | **Hazard ratio (95% CI)** | **P value** |  | **Hazard ratio (95% CI)** | **P value** |
| **Age** | 177 | 1.028 (1.007 - 1.049) | **0.009** |  | 1.020 (0.999 - 1.040) | 0.058 |
| **Gender** | 177 |  | 0.350 |  |  |  |
| Female | 80 | Reference |  |  |  |  |
| Male | 97 | 0.823 (0.548 - 1.238) | 0.350 |  |  |  |
| **Grade** | 173 |  | **0.016** |  |  |  |
| G1 | 30 | Reference |  |  | Reference |  |
| G2 | 95 | 1.988 (1.024 - 3.860) | **0.042** |  | 0.947 (0.474 - 1.892) | 0.877 |
| G3 | 48 | 2.621 (1.302 - 5.275) | **0.007** |  | 0.930 (0.443 - 1.950) | 0.847 |
| **T classification** | 175 |  | **0.015** |  |  |  |
| T1+T2 | 31 | Reference |  |  | Reference |  |
| T3+T4 | 144 | 2.052 (1.088 - 3.869) | **0.026** |  | 1.037 (0.519 - 2.070) | 0.919 |
| **N classification** | 172 |  | **0.002** |  |  |  |
| N0 | 49 | Reference |  |  | Reference |  |
| N1 | 123 | 2.113 (1.258 - 3.547) | **0.005** |  | 1.802 (1.035 - 3.137) | **0.037** |
| **M classification** | 173 |  | 0.980 |  |  |  |
| M0 | 169 | Reference |  |  |  |  |
| M1 | 4 | 0.982 (0.241 - 4.007) | 0.980 |  |  |  |
| **Risk score** | 177 | 3.330 (2.156 - 5.144) | **< 0.001** |  | 3.152 (1.879 - 5.288) | **< 0.001** |

**Table S4** Cox regression of risk score in ICGC cohort

| **Characteristics** | **Total(N)** | **Univariate analysis** | |  | **Multivariate analysis** | |
| --- | --- | --- | --- | --- | --- | --- |
|  |  | **Hazard ratio (95% CI)** | **P value** |  | **Hazard ratio (95% CI)** | **P value** |
| **Age** | 87 | 1.027 (0.998 - 1.057) | 0.072 |  | 1.031 (1.001 - 1.062) | **0.040** |
| **Gender** | 87 |  | 0.749 |  |  |  |
| Female | 41 | Reference |  |  |  |  |
| Male | 46 | 1.091 (0.638 - 1.867) | 0.750 |  |  |  |
| **Grade** | 86 |  | **0.004** |  |  |  |
| G1+G2 | 53 | Reference |  |  | Reference |  |
| G3+G4 | 33 | 2.247 (1.301 - 3.879) | **0.004** |  | 1.717 (0.950 - 3.103) | 0.073 |
| **T classification** | 87 |  | 0.171 |  |  |  |
| T1+T2 | 13 | Reference |  |  |  |  |
| T3+T4 | 74 | 1.692 (0.759 - 3.770) | 0.199 |  |  |  |
| **N classification** | 87 |  | **0.001** |  |  |  |
| N0 | 28 | Reference |  |  | Reference |  |
| N1 | 59 | 2.733 (1.399 - 5.339) | **0.003** |  | 2.610 (1.318 - 5.171) | **0.006** |
| **Risk score** | 87 | 2.879 (1.622 - 5.113) | **< 0.001** |  | 2.192 (1.213 - 3.960) | **0.009** |

**Table S5** Cox regression of PVT1 expression in patient cohort

| Clinicopathological  feature | Univariate analysis | | | Multivariate analysis | | |
| --- | --- | --- | --- | --- | --- | --- |
|  | Risk ratio | 95%CI | P | Risk ratio | 95%CI | P |
| Age  ≥60 vs. <60 | 1.04 | 0.77-1.4 | 0.801 |  |  |  |
| Gender  Female *vs.* male | 1.19 | 0.89-1.59 | 0.248 |  |  |  |
| Diabetes  Yes *vs.* No | 1.13 | 0.73-1.73 | 0.586 |  |  |  |
| CA199  Elevated vs Normal | 0.71 | 0.47-1.08 | 0.113 |  |  |  |
| Tumor size  ≥3cm *vs.* <3cm | 1.12 | 0.83-1.51 | 0.466 |  |  |  |
| Tumor site  Body&tail vs Head | 1.09 | 0.80-1.47 | 0.588 |  |  |  |
| Local invasion  Yes *vs.* No | 1.68 | 1.22-2.33 | **0.002** | 1.73 | 1.16-2.58 | **0.007** |
| Differentiation  poor *vs.* well | 1.5 | 1.08-2.09 | **0.016** | 1.52 | 1.07-2.17 | **0.02** |
| T classification | 1.15 | 0.91-1.45 | 0.249 |  |  |  |
| N classification  N1 *vs.* N0 | 1.65 | 1.21-2.24 | **0.001** | 1.13 | 0.3-4.17 | 0.858 |
| PVT1 expression  High *vs.* Low | 1.78 | 1.32-2.4 | **<0.001** | 1.77 | 1.22-2.56 | **0.003** |

**Table S6** Cox regression of PVT1 expression in TCGA cohort

| Clinicopathological  feature | Univariate analysis | | | Multivariate analysis | | |
| --- | --- | --- | --- | --- | --- | --- |
|  | Risk ratio | 95%CI | P | Risk ratio | 95%CI | P |
| Age  ≥60 vs. <60 | 1.42 | 0.91-2.23 | 0.123 |  |  |  |
| Gender  Female *vs.* male | 0.82 | 0.55-1.24 | 0.3499 |  |  |  |
| Diabetes  Yes *vs.* No | 0.92 | 0.53-1.61 | 0.7767 |  |  |  |
| Chronic pancreatitis  Yes vs No | 1.17 | 0.56-2.45 | 0.6781 |  |  |  |
| T classification | 1.57 | 1.01-2.43 | 0.0439 | 1.09 | 0.6-1.98 | 0.7673 |
| N classification  N1 *vs.* N0 | 2.11 | 1.26-3.55 | 0.0047 | 1.97 | 0.93-4.17 | 0.0748 |
| M classification  M1 vs M0 | 1.05 | 0.25-4.39 | 0.9466 |  |  |  |
| PVT1 expression  High *vs.* Low | 1.56 | 1.04-2.35 | 0.0329 | 1.56 | 1.03-2.38 | **0.0368** |

**Table S7** Sequences of siRNAs used in this study

| **Target** | **Sequence** |
| --- | --- |
| siPVT1-1 | GCTTGGAGGCTGAGGAGTT |
| siPVT1-2 | CCCAACAGGAGGACAGCTT |
| siCDC6-1 | GTGTGAGACTATTCAAGCA |
| siCDC6-2 | GACGAAGATTGGTATTTGA |
| siGBP4-1 | GGACGAAGTGCCAGAAGAA |
| siGBP4-2 | ACAGGAAAATCCTATCTCA |
| siCOL17A1-1 | GAGTGAAATTCGAGTTCGA |
| siCOL17A1-2 | GGCAACCTATGCAGCTGAA |
